# Potential Mechanisms of Influence Between Spiritual Practices and Cognitive Health: A Systematic Review and Conceptual Model

**DOI:** 10.3390/brainsci15121296

**Published:** 2025-11-30

**Authors:** Katherine Carroll Britt, Hayoung Oh, Augustine Cassis Obeng Boateng, Sherry Morgan, Sato Ashida, Corey Nagel, Roland J. Thorpe, Harold G. Koenig

**Affiliations:** 1 College of Nursing, University of Iowa, Iowa City, IA 52242, USA; 2 Department of Sociology, College of Arts & Sciences, Baylor University, Waco, TX 76798, USA; 3 School of Nursing, University of Pennsylvania, Philadelphia, PA 19104, USA; 4 Holman Biotech Commons, University of Pennsylvania, Philadelphia, PA 19104, USA; semorgan@upenn.edu; 5 College of Public Health, University of Iowa, Iowa City, IA 52242, USA; 6 College of Nursing and Public Health, University of Arkansas for Medical Sciences, Little Rock, AR 72205, USA; 7 Department of Health, Behavior, and Society, Johns Hopkins Bloomberg School of Public Health, Baltimore, MD 21205, USA; 8 Departments of Psychiatry and Medicine, Duke University Health System, Durham, NC 27701, USA

**Keywords:** spirituality, religion, memory, executive function, cognition, Alzheimer’s, dementia

## Abstract

**Background/Objectives**: This systematic review summarizes the evidence regarding potential mechanisms underlying the relationship between spiritual practices and cognitive health in adults. The review was performed based on the Preferred Reporting Items for Systematic Reviews and Meta-Analyses (PRISMA) checklist. **Methods**: An extensive search of six electronic databases (i.e., PubMed, PsycINFO, Embase, Sociological Abstracts, ATLA, CINAHL) was conducted using keywords related to spiritual practices, religious activities, and cognitive health from 1 January 2000 to December 2025. The quality of the included studies was assessed using the Mixed Methods Appraisal tool aligned with the study design. **Results**: A total of 34 studies were selected for final inclusion in this review, and a narrative synthesis is provided. The studies were conducted throughout the world, with most studies conducted in North America and Asia. Short- and medium-term effects of spiritual practices on cognitive health were identified across heterogeneous practices ranging from meditation, tai chi and yoga to general spiritual and religious activities. Across the strongest evidence, 73% of the randomized control trials examined reported better cognitive health among the more spiritually active; neurobiological factors of decreased inflammation and improved immune response helped to explain these effects. A total of 5 studies examined mediators, while 29 studies contained only secondary outcomes for determining potential mechanisms. Informed by findings across psychosocial, behavioral, and neurobiological pathways, a conceptual model was created and needs testing. **Conclusions**: Possible mechanisms for spiritual effects on cognition may be through mental health and neurobiological factors, although more rigorous and long-term studies are needed.

## 1. Introduction

Recognized as a public health priority, Alzheimer’s disease and related dementia (AD/ADRD) is one of the costliest chronic health conditions in the world, posing immense economic and social challenges to the healthcare system [1]. It is estimated that more than 55 million individuals are living with AD/ADRD globally, costing an estimated 1.3 trillion US dollars [2]. In addition, people are living longer worldwide, projecting vastly greater costs in the future [3]. With increasing age, an individual’s risk of developing AD/ADRD increases, such that 13.1% of those aged 75 to 84 years and 33.3% of those aged 85 years and older have AD/ADRD [4,5]. With populations growing older and healthcare systems struggling to support those with dementia, identifying resilience factors in cognitive aging is essential to decrease a potentially catastrophic burden on society in the future.

As the aging population continues to increase, so does its diversity. In the U.S., approximately 45% of those 65 years and older in 2060 will be part of a minority (non-White) ethno-racial group [6]. Due to persistent social and health inequalities, Black and Hispanic populations have the highest prevalence of AD/ADRD [6,7,8]. It is projected that the prevalence of AD/ADRD in Black and Hispanic populations in the US will more than quadruple, increasing from 1,003,000 in 2014 to 4,276,000 people with AD/ADRD in 2050 [7]. Furthermore, research has shown that Black and Hispanic populations experience worse cognitive functioning in later life, a significant predictor of AD/ADRD, indicating racial/ethnic cognitive disparities [9,10,11].

Without a cure for the disease, much focus is on interventions to modify risk factors for AD/ADRD. These risk factors include less education, hypertension, obesity, smoking, physical inactivity, diabetes, hearing loss, depression, social isolation, excessive alcohol consumption, traumatic brain injury, air pollution, social isolation, vision loss, and high LDL cholesterol [12]. Since many studies report the importance of social connectedness in cognitive health, one psychosocial factor appears to influence several risk factors, creating a possible target for intervention—spirituality.

Spirituality includes culture-specific beliefs and practices (may include religious involvement for some) within the context of trusted social communities, which is especially true for coping with stress among historically underrepresented populations experiencing inequalities [13]. Spirituality appears to contribute to better cognitive function in many studies [14,15,16,17,18,19,20,21,22,23,24,25,26,27,28], resilience [29,30,31], and better brain health [23,32,33]. In support of these findings, individuals who engage in more socially active lifestyles exhibit better cognitive function [34,35]. Spiritual practices may serve as a lifestyle resource in this regard, contributing to cognitive reserve [36,37,38,39] by providing a cognitively stimulating activity, increasing sensory stimulation, reducing stress, and acting through other psychological pathways, resulting in more efficient brain network utilization [40,41,42]. More broadly, spiritual practices may include any activities or practices that provide meaning, reflection, and personal connection with something beyond the physical realm. This understanding is based on a definition coined by experts in religion, spirituality, and health research and clinical practice at a consensus conference intended to define spirituality in research [43]. As a low-cost, easily accessible resource supporting cognitive health, culturally sensitive spiritual practices have the potential to optimize cognitive aging and reduce racial/ethnic cognitive disparities.

**Figure 1:**
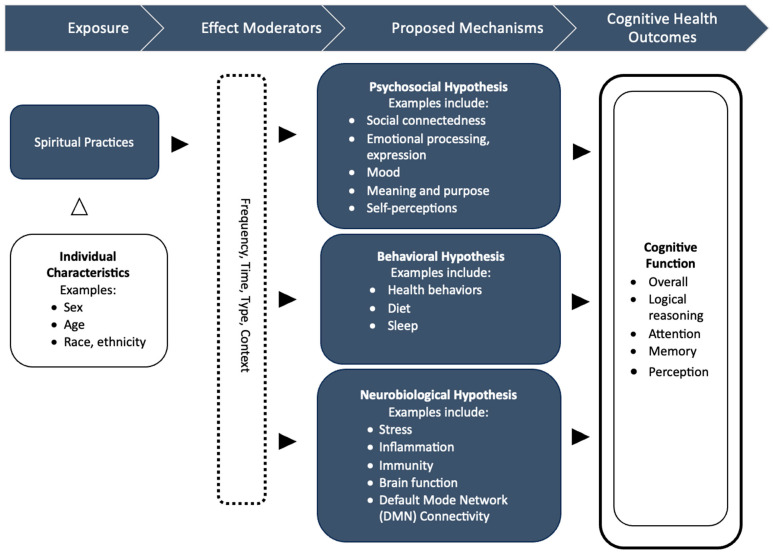
A conceptual model for the influence of spiritual practices on cognitive health outcomes in adults across three pathways: psychosocial hypothesis, behavioral hypothesis, and neurobiological hypothesis.

Despite increasing studies reporting the cognitive health benefits of spiritual practices [21,22,26,44], the underlying mechanisms responsible for these positive effects have not been thoroughly explored [45,46]. In addition, many studies have limited design rigor, with many reporting correlational findings as opposed to causal. Identifying the underlying mechanisms of influence may facilitate the development and delivery of successful interventions that are culturally congruent and reduce disparities. The goals of this article are to: (1) conduct a systematic review of studies on spiritual practices examining the impact on cognitive health outcomes with potential mechanisms, and (2) develop a conceptual model for explaining the influence of spiritual practices on cognitive health outcomes in adults. We start out with a preliminary conceptual model based on the current state of knowledge in cognitive health, with an explanation of three potential broad categories of mechanisms (psychosocial, behavioral, and neurobiological) [47], summarized in Figure 1. Afterwards, we conduct a systematic literature review followed by the presentation of a developed model informed by the review’s results. By summarizing these studies, we examine the extent to which there is an understanding of the pathways and identify where the field needs to go in the future.

### 1.1. Conceptual Model Components

Several possible mechanisms may help to explain how spiritual practices influence cognitive health. First, we review background knowledge on published research focused on spiritual practices and general health. These include reductions in stress, anxiety, and depression; increased social connectedness; stimulation of positive emotions; improved healthy behaviors; increased sleep; reduced inflammation; and increased immunity (see Figure 1). Many of these underlying pathways are interconnected. Below, we discuss the relevant literature to create a clearer picture of the mechanisms illustrated in this conceptual model.

#### 1.1.1. Psychosocial Mechanisms

The first mechanisms relevant to the link between AD/ADRD and spiritual practices are psychosocial mechanisms, which constitute measures of psychological factors, social factors, or both. While there are many psychosocial measures to consider, these three stand out based on published studies: (1) social connectedness, (2) positive emotions, and (3) perceived stress, anxiety, and/or depression.

These three pathways, while not exhaustive, offer a preliminary framework for understanding the most important impact of psychosocial mechanisms. For example, a key aspect of social connectedness is that it captures the interplay between the individual and the community. This is particularly important since group participation and time spent with others are an integral aspect of spiritual practices and are a protective factor against AD/ADRD. First, positive emotions in the context of spiritual practices may serve as a protective factor against AD/ADRD. Second, anxiety and depression are considered risk factors for decline in cognitive health, whereas spiritual practices often protect against such outcomes. We discuss the literature surrounding these psychosocial effects below.

A. Social Connectedness

Individuals’ reports of social support are often associated with activities, beliefs, and community participation. Spirituality is partly defined by connectedness—this can include self, others, nature, or the sacred. Spiritual practices can increase one’s social connectedness. A systematic review of the literature [48] reported significant positive associations between spiritual practices and social support in 82% of 61 studies, and no significant inverse relationships were found. For older adults, the most common form of social support reported was from religious organizations. As individuals age, their social network grows smaller [49]. Since more studies are showing that loneliness and social isolation contribute to poor cognitive health outcomes, maintaining social connectedness is essential for older adults [42,50,51,52,53]. Spiritual practices contribute to an individual’s spiritual well-being, which in turn is associated with social support and connectedness [54]. Spiritual practices are also related to social capital, which is an indicator of community health and support [48].

Social support may consist of emotional support or involve the sharing of practical resources (instrumental support). Studies among African American participants found that spiritual and religious social support was associated with fewer depressive symptoms [55] and less psychological distress [13]. Social support has also been found to be a mediating factor between prayer/meditation and life satisfaction [56]. Spiritual practices are associated with greater social support and more extensive social networks, and by reducing loneliness and social isolation, they may contribute to better cognitive health [57].

B. Positive Psychological Emotions

Positive emotions are associated with better cognitive functioning and reduced risk of AD/ADRD. For example, meaning and purpose in life (MPIL) is associated with slower rates of cognitive decline and decreased risk of mild cognitive impairment (MCI) and AD/ADRD [58,59], thereby protecting against changes in brain function [60]. Individuals with greater MPIL may engage in healthier lifestyles that support cognitive function [61]. Since biomarkers such as systemic inflammation and cellular stress response are associated with cognitive health [62,63,64], and MPIL is associated with lower interleukin-6 (IL-6) plasma levels [65], lower hemoglobin A1c (HbA1c) levels [66], and lower cortisol levels [67], it stands to reason that MPIL would be associated with better cognitive functioning and slower cognitive decline.

Optimism is also associated with a decreased risk of cognitive impairment in older adults [68]. The degree to which a person experiences pleasant moods, such as joy or enthusiasm, is termed positive affect, which is also associated with better memory [69,70] and greater global cognitive function [71]. Positive affect increases the likelihood of adaptive and healthy behaviors, attitudes [72], and social relationships [73]. Optimism may also affect cognitive health by increasing motivation to work through challenging situations. In an 8-year longitudinal study, higher self and spouse optimism was associated with better cognitive functioning [74]. Thus, there is ample evidence that positive attitudes and emotions may enhance cognitive health [58].

C. Perceived Stress, Anxiety, and/or Depression

In contrast, greater levels of perceived stress, anxiety, and depression are linked to poorer cognitive health and may help alleviate mental health problems like these. For example, participants in a longitudinal study who reported spirituality as highly important at baseline were 76% less likely to experience a major depressive episode during follow-up [75]. Furthermore, spiritual well-being is associated with fewer symptoms of depression and anxiety [23,76]. Likewise, higher levels of spirituality (prayer, religious coping, religious attendance) among community-dwelling African American adults in the U.S. were associated with better cardiovascular health [77], which is often connected with cognitive functioning. How much strain a person perceives when demands exceed their capacity to adapt [78] appears to be related to cognitive health as well. Investigators [79] have reported that perceived stress was associated with lower cognitive scores and a faster rate of cognitive decline over time. Perceived stress influences inflammatory and hormonal markers of brain aging, which is thought to be the mechanism by which it contributes to cognitive decline [80].

#### 1.1.2. Behavioral Mechanisms

The second mechanism relevant to the link between AD/ADRD and spiritual practices is behavioral. This involves how an individual’s actions through their spiritual practices may affect their cognitive health. Behavioral mechanisms may be categorized into (1) healthy behaviors and (2) sleep. Healthy behavior is a rather all-encompassing term, although the term is primarily limited to drug/alcohol/cigarette use, exercise, and diet. For the current systematic review, the studies reviewed are not equally distributed among these three aspects of healthy behavior. Furthermore, some studies, such as yoga and tai chi, require movement of the body, yet researchers have not attempted to separate yoga into a spiritual practice or an exercise. Rather, it has been defined as simply an exposure.

Much of the literature has suggested that sleep influences the risk for AD/ADRD. Specific beliefs about God, such as those involving divine control or spiritual practices, have been associated with both the quantity and quality of sleep and, consequently, may act as a protective factor for AD/ADRD through this pathway. We discuss more on the literature surrounding these behavioral factors below.

A. Healthy Behaviors

Spiritual practices positively impact health behaviors such as diet, exercise, cigarette smoking, excessive alcohol consumption, and illicit drug use [23,81]. Individuals who engage in spiritual and religious activities report lower alcohol use [82], less smoking, and fewer risky social behaviors [48]. Individuals with spiritual beliefs report higher fruit and vegetable consumption and lower alcohol consumption compared to others [83]. Those scoring higher on spiritual measures are more likely to emphasize respect for the body and less likely to engage in risky behaviors. In a Danish study [84], individuals using prayer and meditation had healthier dietary patterns and were less overweight. In addition, those who attended religious services more frequently (i.e., attending church, mosque) also had healthier dietary practices and were less likely to be smokers. These findings are supported by additional studies of individuals participating in spiritual or religious activities who report healthier lifestyles [23,85,86,87]. More regular engagement in spiritual and religious practices may provide a supportive community, decrease stress, and lead to less risky health behaviors. This could be due to embedded values and norms in specific spiritual and religious traditions that promote healthier lifestyles [84].

In a study among predominantly Hispanic adults, individuals who reported being spiritual and religious had lower substance use (i.e., alcohol and tobacco) than those who indicated they were neither spiritual nor religious [88]. Other studies have reported that young people who have an active spiritual life, as reflected by involvement in a faith community and prayer, are less likely to use drugs and alcohol [89,90,91,92]. In addition, a two-year study [93] reported that young people who never attended religious services, compared to those who attended once a week, were twice as likely to use alcohol and tobacco and four times as likely to use illicit drugs. As excessive alcohol consumption and smoking are behaviors that increase an individual’s risk for AD/ADRD, these are important considerations [12]. Spiritual practices may enhance self-esteem, moral values, and coping skills and thus reduce the need to turn to substances (i.e., tobacco, alcohol, recreational drugs) in the face of stress [23,94].

B. Sleep

Sleep is essential for learning, memory, and cognitive processing. Poor sleep may contribute to worse cognitive functioning, increasing one’s risk of developing dementia [95]. Spirituality may improve sleep among adults by promoting relaxation, well-being, and a sense of purpose and meaning while decreasing psychological distress and physical stress. Adults who attended religious services more than once per week were more likely to report better sleep quality than those who never attended religious services or attended less than once per month [96]. Frequency of prayer was also associated with better overall sleep quality.

In comparison, religious doubts or struggles have been associated with poorer sleep quality and use of sleep medications [97]. Mindfulness meditation interventions have also been shown to improve sleep quality [98]. As stressful life events are associated with worse sleep quality, spiritual and religious beliefs have been shown to buffer the negative effects of life trauma on sleep [99]. The framing of situations using spiritual and religious cognitions, such as secure attachment to God and assurance of salvation, may diminish the effect of stressful life events on sleep quality [97]. These spiritual and religious beliefs about health and well-being may shape one’s interpretation of stressors, thereby reducing distress that keeps people awake [100]. Certain events may be viewed as less threatening based on beliefs about self and reality [101]. Another recent study [102] found that belief in divine control (belief in God’s authority in the universe) and religious service attendance were associated with better sleep quality. Further, Castro and colleagues [103] report that spiritual well-being was associated with better sleep quality among adult patients with chronic disease. These findings indicate that spirituality and religion contribute to better sleep quality by supporting greater spiritual well-being and promoting meaning and life purpose through situation framing.

#### 1.1.3. Neurobiological Mechanisms

The third category of mechanisms considered in this review is neurobiological mechanisms. These are brain and biological processes that involve measures of biochemicals. Specifically, we examine (1) stress, (2) neurological aspects, and (3) inflammation and immunity.

Situations that increase stress may be related to external events such as discrimination of marginalized populations or stress from internal issues such as the diagnosis of a serious illness [104]. In both cases, spirituality may be a key protective factor in reducing stress. Studies that have examined the neurological components of spirituality have found that spiritual interventions such as going on a spiritual retreat or engaging in prayer have been positively associated with increased brain stimulation and formation of white matter [see below]. Lastly, spirituality can be an effective way to decrease inflammation through specific immune pathways like pro-inflammatory markers and cytokines [see below].

A. Stress

Individuals may turn to spirituality when facing stressful situations, such as discrimination, serious illness, or death [105]. For instance, marginalized populations such as non-Hispanic Black and Hispanic/Latinx populations with MCI and dementia report higher importance of spirituality and frequency of private prayer than White/Caucasian populations do [16,18].

Spirituality may provide psychological resources to assist in coping and adaptation to stressful situations. Some spiritual traditions emphasize forgiveness, compassion, and humility; these practices may help negate the influence of stress on health outcomes, as spiritual practices are an effective mechanism for coping. Indeed, individuals engaging in spiritual practices report less stress [23,106], and spiritual practices may improve coping by impacting the stress appraisal process [107]. For example, in a functional magnetic resonance imaging (fMRI) study, spiritual practices attenuated neural responses to stress reactions, supporting emotion regulation during stress exposure [108]. Mindfulness stress reduction practices have also been associated with less depression and greater spiritual well-being in participants with dementia [109].

B. Neurobiology (brain function, white matter, etc.)

Whether due to aging, trauma, depression, or other life-course events, spirituality can impact both the formation of the physical brain and affect other cognitive functions. For example, spirituality is also known to protect brain function in terms of overall brain functional connectivity [110], thickness of brain cortices [33], and amount of white matter [111].

The protective effects of spirituality on brain neurobiology have important health implications for the general public. A 2022 systematic review [112] examined the neuroscience of spirituality, finding that neurological effects were particularly important in protecting against depression, alcohol/substance use, and mental health. Researchers found that spiritual and religious practices were protective against familial risk for depression by affecting the thickness of cerebral cortices, preventing the thinning of cortices, increasing white matter, and expanding pial surfaces. Another study [113] found that the self-reported importance of spirituality and religion was associated with decreased default mode network (DMN) connectivity in the left parietal lobe, which may help to explain protective effects from depression through attention, emotion regulation, and difficulty concentrating. For substance use, one study [114] mentioned in the review found that prayer may reduce cravings for alcohol by increasing the neural processes related to attention and control.

C. Inflammation and Immunity

Since emotion regulation is associated with inflammation [115], individuals who engage in spiritual practices may have lower inflammation due to effects on the regulation of emotions in stress response [108]. Spiritual practices may influence cognitive health by downregulating central inflammatory pathways and improving immune regulation, in which psychological stress plays a role [116,117,118,119]. A systematic review of randomized controlled trials reported that mindfulness meditation was associated with changes in immune system processes involved in biological aging, inflammation, and immunity [120]. Findings from this review suggested mindfulness meditation may enhance a person’s immune defenses to protect against viral and bacterial infection and age-related disease (i.e., cardiovascular disease, frailty, etc.). Another recent study reported that mindfulness training reduced systemic markers of inflammation measured by C-reactive protein (CRP) in midlife-to-older adults at particular risk for systemic inflammation [121].

In another review, Falkenberg et al. [122] found that mind–body practices such as yoga may downregulate pro-inflammatory markers such as Interleukin (IL) 1beta, IL-6, and TNF-alpha. Estevao [123] found that yoga had a beneficial effect on circulating cortisol, inflammatory markers (CRP), and cytokines (IL-1beta, IL-6, TNF-alpha, interferon-gamma (INF-γ), suggesting that mind–body practices may help to manage stress and reduce depression through regulation of vascular and immune functions. In addition, a high frequency of prayer in advanced cancer patients was associated with decreased inflammation measured by white blood cells and CRP, which in turn was associated with improved survival [124]. Finally, a higher frequency of religious service attendance in older adults was associated with fewer impairments in activities of daily living and instrumental activities of daily living, mobility limitations, and lower levels of inflammation (IL-6) and coagulation parameters (soluble vascular cell adhesion molecule, D-dimer) [125].

#### 1.1.4. Summary of Conceptual Model

Several studies have reported a positive association between spiritual practices and cognitive health [14,19,20,21,22,23,24,25,26,27,28]. As discussed above, the possible mechanisms underlying these associations may be categorized into three domains: psychosocial, behavioral, and neurobiological. These factors are summarized in the conceptual model (Figure 1). To our knowledge, the present article is the first systematic review of the mechanisms responsible for the influence of spiritual practices on cognitive health.

Objective: To present a conceptual model identifying possible mechanisms to explain the influence of spiritual practices on cognitive health and to conduct a systematic review of the evidence to inform this model.

## 2. Materials and Methods

We used the Preferred Reporting Items for Systematic Reviews and Meta-analysis (PRISMA) checklist as a guide to conduct and report the findings of this review (See Supplemental Table S1). This review was not previously registered in PROSPERO (York, UK).

### 2.1. Search Strategy

Six electronic databases (PubMed, PsycINFO, Embase, Sociological Abstracts, ATLA, CINAHL) were searched from 1 January 2000 to December 2023 using combinations of official vocabulary and keywords representing common terminology of spirituality, religion, and cognition; Table 1 shows the PubMed search strategy employed. To capture therapies that are spiritually or religiously based, we searched PubMed using MeSH terms and keywords focusing on those concepts. For example, “Spiritual Therapies” [MeSH] “explodes” to collect several other MeSH terms that are more specific; one of them is “Meditation” [MeSH]. After all terms for each concept were searched and combined, the addition of the truncated keyword terms “spirit *” or religio * was added to ensure that this focus of the research would be included; for example, this would exclude articles on meditation that did not focus on spirit * or religio *. We used the PubMed search as the model for searching the additional 5 databases (see Supplementary Table S2). Strategies were adapted as needed. This followed other published literature review strategies that are also focused on spirituality and cognitive health [17,126]. To minimize the inclusion of the narrower interpretation of spirituality more common in older publications and to align with other published literature reviews on spiritual practices [17,127], the initial year chosen for the search was 2000. The search included a review of reference lists of recent publications on spiritual practices and cognitive health outcomes. The authors conducted an additional review (June 2025), repeating the steps above, and searched and reviewed more recent articles published from January 2024 to December 2025, which provided an additional n = 566 articles.

### 2.2. Data Extraction

Two of three authors (KCB, HO, ACOB) independently reviewed full-text articles using Covidence software (Melbourne, AUS) for the literature review and extracted key information into a Microsoft Excel file for organization. Authors met frequently to discuss extracted data, organization, and completion; the author (KCB) reviewed all articles and table extractions, confirming the data. To guide the collection of study characteristics, the following study data were extracted from each article: first author, publication year, study design, study location, sample size, demographic characteristics, spiritual practice or intervention type, cognitive function or domain measurement, mediators and secondary outcomes and measures, and main findings. Any discrepancies among decisions were resolved through discussion by the third author, not involved in the original review (KCB, HO, or ACOB).

### 2.3. Eligibility Criteria

1. Original research published in English focused on spiritual practices and cognitive health, with potential mechanisms examined.
2. Type of participants. Participants were adults aged 18 and older at baseline. Studies targeting populations with psychiatric disorders (i.e., schizophrenia) were not included.
3. Exposure. Types of studies: Any community, hospital, hospice, palliative care, assisted living, or long-term care-based spiritual practice or spiritual intervention was included.
4. Outcome. Studies were included if they reported statistical analyses of cognitive function or a cognitive domain.
5. Mediators. Studies were included if they reported statistical analyses of potential psychosocial, behavioral, and neurobiological mechanisms as mediators or secondary outcomes.
6. Study designs. Study designs included cross-sectional, longitudinal studies, such as observational, experimental, or quasi-experimental studies of at least one week in duration. Case studies, abstracts only, dissertations, theses, qualitative studies, and gray literature were excluded.

### 2.4. Methodological Assessment

The Mixed Methods Appraisal Tool (MMAT) [128] was used to assess study quality and potential bias. Matching study design to the quality appraisal MMAT, three authors (KCB, HO, ACOB) independently evaluated the methodological quality and risk of bias for included studies. A percentage was given for each study based on the quality appraisal tool, representing the study’s fulfillment of the quality criteria (5 items), representing various sources of rigor and potential bias in scientific research (see Table 2). These studies were then categorized as high (≥80%), medium (60–80%), or low (≤40%) quality, based on the percentage of items addressed. Studies were not excluded based on quality.

## 3. Results

### 3.1. Study Selection

**Figure 2:**
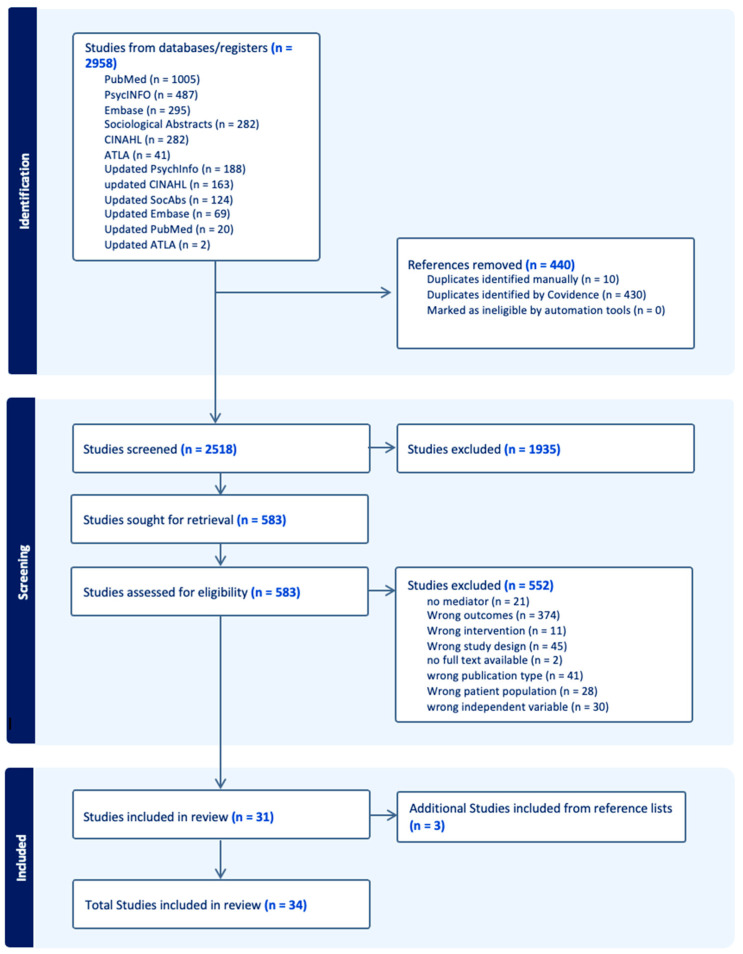
The PRISMA Figure of searched articles with excluded and included studies.

In this systematic review, 2958 studies were obtained using database searches (see Figure 2). A total of 440 duplicates were removed. After a comprehensive review of titles and abstracts, 1935 studies were removed due to non-alignment with the review purpose, and 552 studies were removed due to no mediator or secondary outcome, wrong outcome, intervention, study design, publication type, patient population, independent variable, or no full text available. An additional three studies were identified in the reference lists for inclusion, making the total number of included studies equal to 34.

### 3.2. Study Characteristics

These 34 studies were conducted around the world, with the majority in North America [15,16,18,129,130,131,132,133,134,135,136,137,138,139,140,141,142] and across Asia [143,144,145,146,147,148,149,150,151,152,153]. In addition, three studies were conducted in Europe [154,155,156], two studies in South America [157,158], and one study in Australia [159].

In total, 20 studies used a randomized controlled trial (RCT) design [130,132,133,134,135,137,138,140,142,144,145,146,147,148,149,150,152,154,156,159]. A total of eight studies were observational. Five were cross-sectional [15,16,18,143,158] and two were longitudinal [131,155]. Only one was a descriptive intervention study [141], and the remaining six studies used a quasi-experimental design [129,136,139,151,153,157].

Only five studies examined mediating factors across various statistical approaches [131,133,137,144,158], with the remaining studies examining secondary outcomes [15,16,18,129,130,132,134,135,136,138,139,140,141,142,143,145,146,147,148,149,150,151,152,153,154,155,156,157,159]. Studies were organized according to spiritual practice category and study design to synthesize findings.

#### 3.2.1. Meditation Studies

Of the included studies focused on meditation activities (n = 13) (see Table 3), five studies used a quasi-experimental design [129,136,139,151,157], one was a descriptive intervention study [141], six were randomized control trials [134,135,137,138,147,159], and one was a longitudinal observational study [131].

Ten out of the thirteen studies reported improved cognition among participants who engaged in meditative practices compared to control groups: positive cognitive skills and DNA methylation [129,131]; improved episodic memory but not executive function [141]; improved ability to shift attention, attention selection, concentration, and accuracy [151]; improved IQ and memory [139]; improved verbal fluency and logical memory test [159]; improved perceived cognitive impairment and perceived cognitive abilities [134]; greater gains in cognitive function among meditation group [135]; improved cognition and executive function [136]; improved memory and improved memory quotient [147].

Sex differences in cognitive performance were noted in one study [138], with women outperforming men on memory, cognitive, and speed processing tasks. In addition, they found significant differences in cognitive performance between meditation and control groups but observed no significant difference between the two meditation groups (8-week vs. 12-week programs). Notably, two studies [137,157] found that meditation did not enhance cognitive function. Lenze et al. (2022) [137] reported no significant improvements in episodic memory or executive function following mindfulness training, exercise, or a combination of both. Additionally, no improvement was observed in executive function or social cognition performance in either the control or meditation groups.

Two studies that examined potential mechanisms by which meditation may impact cognition [131,137] did not find significance. Bhattacharyya et al. (2023) [131] examined self-esteem, while Lenze (2022) [137] examined multiple factors: aerobic fitness, insulin insensitivity and resistance, body fat and fat-free masses, physical performance, plasma cortisol levels, physical activity, sleep, and mindfulness state.

Among reported secondary outcomes, participants in the meditation group showed alterations in gene regulation through DNA methylation [129], a reduction in psychological stress response [134,136,141,147,151], reductions in cortisol levels [147], enhanced quality of life [134,159], improved mood [134], increased mindfulness [136], increased cerebral blood flow [139], improvement in telomerase activity [81], and lower CRP levels compared to control groups [159]. Additionally, they experienced reduced anxiety and depression levels [157], increased spiritual well-being, and better mental health (i.e., fewer depressive symptoms and better mental health [135,138]) relative to controls.

#### 3.2.2. Yoga Studies

Of the included studies focused on yoga activities (n = 8) (see Table 4), all eight studies were randomized controlled trials. Three of eight studies specified yoga generally [130,142,154], but the other five studies specified the specific type of yoga, such as Hatha yoga [140], Yogic relaxation [149], Kundalini [132,133], and Sahaj yoga [150]. Studies using control groups were also diverse, with three studies having a control group with no yoga (normal daily routines) [130,149,154], two studies using memory enhancement training [132,133], one study with anti-depressant medication [150], and one study with healthy participants [142]. At 12 weeks and 24 weeks, both Kundalini yoga and memory enhancement training groups showed significant memory improvement; however, only Kundalini yoga showed significant improvement in executive functioning. Only the Kundalini yoga group showed significant improvement in depressive symptoms and resilience at week 12, and one study with a walking class [140].

Five of the eight studies reported that those in the yoga groups improved some aspect of cognitive health compared to the control, including letter cancelation [150], visual–spatial skills [142], improved overall cognitive scores analyses [130,133], and improvements in memory [132]. The three other studies reported that there was no statistical difference between groups [140,149,154].

For secondary outcomes, three studies showed that those in the treatment group of yoga showed reductions in depressive and/or perceived stress symptoms [132,142,150]. The Grzenda et al. study [133] was particularly unique. Researchers found that those who participated in yoga were able to uniquely change certain pathways that such as interferon gamma and other psycho-neuro-immune pathways that are associated with aging. Furthermore, aging markers increased over time in the control group but not in the yoga treatment group.

#### 3.2.3. Tai Chi Studies

Out of the 34 included studies, 7 studies examined tai chi (see Table 5). Of these seven tai chi studies, one study [158] was observational and cross-sectional, and six studies [144,145,146,148,152,156] were randomized controlled trials. Six out of the seven tai chi studies reported better cognitive health in the tai chi groups included improved cognitive function [144,145,146,148] attention/processing speed/executive function [144,148,152], executive control [144], delayed recall [143], memory [144,152], visuospatial processing and accuracy in response inhibition [156], reduced time to complete cognitive testing [145], and lower risk of developing dementia [146]. One study [158], which was a case–control design, found no significant differences in cognition in the tai chi group compared to the control group [158].

As for potential mechanisms, one study [158] reported specific brain region activation/responses, investigating neural correlates of cognitive processes during tai chi, and found that the tai chi participants had less brain activation in regions related to working-memory-, visual-, and language-related regions compared to the water aerobics group, possibly reflecting greater brain efficiency to perform these cognitive tasks—however, this study did not see significant differences in cognition between the tai chi and water aerobics groups. The other studies identified secondary outcomes. Two studies [152,156] examined neuropeptides, reporting that the tai chi groups had increased synaptic plasticity in the brain. One study [156] reported improved reaction time in mental shifting in the tai chi group. One study [158] found the tai chi group had lower anxiety, and two studies [146,156] reported lower depression scores. One study [148] found that the tai chi group had better sleep. One study [156] reported decreased perceived stress. Chen et al. [144] found improved fasting glucose levels, glycation end-products, and their receptors. In addition, four studies found greater functional health [145,156] and functional balance [146,148].

#### 3.2.4. General Religious and Spiritual Activities

Out of the 34 included studies, 6 examined various general religious and spiritual activities (i.e., private prayers and fasting, religious service attendance, religiosity, spirituality, music intervention) (see Table 6). Most of these studies were observational (n = 5/6). Four were cross-sectional [15,16,143] and one was longitudinal [155]. One study [153] was quasi-experimental.

All six studies [15,16,18,143,153,155] reported better cognitive function in participants with higher religious and spiritual activities. Secondary outcomes of these studies include improved quality of life, lower depression, and higher functional health [143]. Coin et al. [155] reported significant functional decline in both the high and low religiosity groups, but the high-religiosity group had less functional decline compared to the low-religiosity group. Other studies found fewer behavioral symptoms [15,16,18], and two studies [16,18] found fewer sleep disturbances, but mixed results were shown in a third study [15] with fewer sleep disturbances at one time point but higher sleep disturbances at the second time point. No differences in depressive symptoms were reported in the Tai et al. [153] study. No mediating factors were examined.

### 3.3. Summary Comparison of Mechanistic Pathways

The following Table 7 visually summarizes the narrative descriptions above across mechanistic pathways and cognitive outcomes in an organized table (see Table 6).

### 3.4. Heterogeneity of Spiritual Practices

Importantly, there was moderate to significant heterogeneity within some of the spiritual practices. Within general religious and spiritual interventions, participation varied from attending religious services to praying to listening to music. While mediation was more consistent, some studies utilized meditative relaxation techniques compared to a Tibetan sound meditation. We found that studies examining Tai Chi did not differ significantly in their conception of the spiritual practice.

For yoga, there was considerable heterogeneity. In the eight yoga studies, two studies utilized Kundalini yoga [132,133], which involved chanting, repetitive finger movements, and visualizations. In contrast, this other yoga study [137] primarily focused on specific yoga poses, such as cat-cow pose or cobra pose. However, another study combined both the pose and other more spiritually focused activities, including Himalayan kriya breathing exercises [151]. It is possible, especially in the studies that utilized yoga for its physical poses rather than its more spiritual origins, that individuals who participate in these interventions do not necessarily perceive the yoga to be spiritual but rather as a form of exercise.

### 3.5. Duration of Studies

Study duration generally followed study designs, with cross-sectional studies having the shortest duration [15,16,18,143,158], and the longest were longitudinal studies that extended to two waves of data [131,155]. Besides the two exploratory quasi-experimental or RCT studies that had follow-up within a single day [147,157], most of the studies (many of which were RCTs) had short follow-ups from 4 to 10 weeks [27,129,130,134,135,138,139,149,150,151,154,156,159]. Other studies had longer follow-ups from 4 to 6 months [104,132,133,137,141,142,144,148,152,153], and three studies were one year or longer (12–18 months) [136,145,146].

### 3.6. Study Population Variability

Although beyond the scope of this review, there are several notable variations in the populations studied across and within the various spiritual practices. First and foremost, across all studies, the most dominant study population was older adults, most obviously because cognitive health increases alongside biological age. From this population, some older adults were already diagnosed with dementia or mild cognitive impairment.

However, the meditation interventions were the most diverse in the study population, ranging from cancer patients to college students to family dementia caregivers. Given that nearly all the meditation interventions were positively associated with maintaining or increasing cognitive health, we argue that the heterogeneity of study populations for meditation interventions is a strength, not a limitation. Furthermore, it may also reveal the utility and widespread acceptance of meditation as a spiritual practice across society.

### 3.7. Quality Ratings

The overall ratings for each study are in Table 2. Studies were evaluated based on the study design provided by the MMAT, a critical appraisal tool that includes quality and bias evaluation across studies with multiple designs. Evaluating study quality according to design using this tool allows consideration of differing components and helps account for heterogeneity. Most studies were rated as high quality (27/34), while 5/34 were rated as medium quality, and 2/24 were rated as lower quality.

### 3.8. Summary of Potential Mechanisms & Created Conceptual Model

The strongest evidence is from the intervention studies. Approximately 16 out of the 22 RCT studies (73%) reported improved cognitive health across meditation (n = 5/6) [134,135,138,147,159], yoga (n = 5/8) [130,132,133,142,150], and tai chi studies (n = 6/6) [144,145,146,148,152,156]; no RCTs were conducted for the general religious and spiritual studies. Identified mediators included reduced inflammation and improved immune response in the neurobiological domain. Secondary outcomes included reduced depressive symptoms, perceived stress, stress, systemic inflammation, psychological distress, improved mood, QOL, sleep, self-esteem, functional health and balance, glycation, synaptic plasticity, telomerase activity, and reaction time in mental shifting.

**Figure 3:**
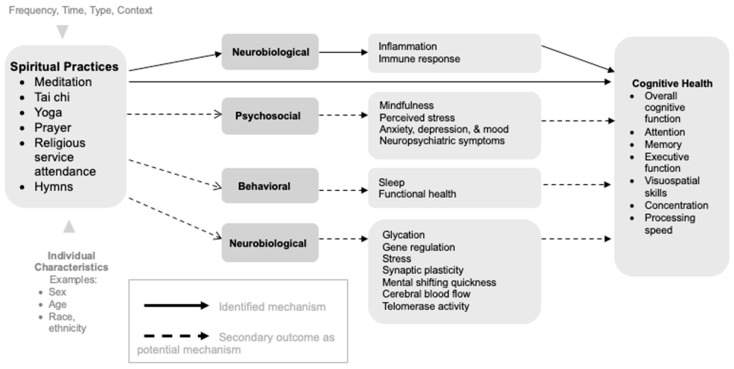
A developed conceptual model of the underlying mechanisms between spiritual practices and cognitive health. The association between spiritual practices and cognitive health via neurobiological pathways shows a solid arrow, while potential psychosocial, behavioral, and additional neurobiological mechanisms show a dotted arrow.

Potential mechanisms from the included studies have been summarized (see Table 8) and formed into a conceptual model (see Figure 3). In this model, we list the various potential mechanisms as mediating factors examined in the studies and secondary outcomes that need further testing as potential mediators of the relationship between spiritual practices and cognitive health. For the five studies that examined mediating factors, only one found significant variables that fall under the neurobiological domain. An RCT examining Kundalini yoga reported reduced inflammatory markers and improved immune response [133]. The other three studies [131,137,144] reported no significant changes in the psychosocial, behavioral, and neurobiological mediating factors they examined. Additionally, one case–control study did not find significant differences in cognition between the two groups (e.g., tai chi vs. water aerobics) [158], though they reported greater brain efficiency in the tai chi group as a potential mechanism for future consideration.

The remaining studies reported secondary outcomes that could serve as potential mediators and need additional testing. These were categorized into the three domains of psychosocial, behavioral, and neurobiological. Under the psychosocial domain, eight studies found decreased or improved depression/mental health symptoms [132,138,142,143,146,150,156,157]; three studies found decreased anxiety or worry severity [136,157,158]; three studies found improved quality of life [134,143,159]; one study found increased mindfulness [136]; and four studies reported decreased perceived/subjective stress in participants engaging in spiritual practices [132,141,142,156].

Under the behavioral domain, five studies found better functional health/activities of daily living [143,145,146,148,156]; four studies found better sleep [16,18,134,148]; and four studies found fewer neuropsychiatric and behavioral symptoms or better mood [15,16,18,134].

In addition to the neurobiological domain components mentioned above as mediators, secondary outcomes under this domain include gene regulation expression [129], decreased stress [134,147,151], improved fasting glucose levels and glycation end-products [144], telomerase activity [135], and lower inflammatory marker levels [159]. Two studies reported increased synaptic plasticity [152,156], while one study reported improved mental shifting reaction time [156] and one study reported increased cerebral blood flow [139].

## 4. Discussion

Findings from this systematic literature review, which examined the impact of broad spiritual practices on cognitive function, contribute to our understanding of how spiritual practices may affect cognitive health. Our findings support neurobiological factors as mediating factors with the strongest evidence from an RCT. Factors for consideration that need future testing as mediators, highlighted here, were identified through causation and association studies, which we differentiate below. These additional secondary outcomes are across psychosocial, behavioral, and neurobiological domains. We break down the findings into these three domains for further discussion below. (Please see Supplementary Table S3 for breakdown by spiritual practice).

### 4.1. Psychosocial Mechanisms

Across all included studies, secondary outcomes in the psychosocial domain reported from RCTs centered around mental health focused on reduced depression, mood, perceived stress, and improved quality of life and mindfulness. Three of these factors are known to be associated with cognitive health. Depression is related to cognitive health as studies report that greater depressive symptoms are associated with greater memory change over time, suggesting psychological mood and memory performance are interrelated [160]. Depressive symptoms appear to be an early sign or risk factor of dementia [161,162]. Research suggests that greater perceived stress is associated with a decline in global cognition, memory, and visuospatial ability among older adults without dementia [80]. Perceived stress was the most reported psychosocial secondary outcome in our review. Further, Zhu et al. [163] suggest that perceived stress influences cognitive function through personality traits of neuroticism and conscientiousness. The second factor of mindfulness, known as focusing on the present moment and acknowledging/accepting one’s feelings and thoughts, appears to support cognitive health through fostering awareness of automatic actions, improving cognitive capacity through working memory, as well as cognitive flexibility [164]. Spiritual practices may decrease perceived stress and prompt mindfulness, which in turn improves cognitive health.

In the other included non-RCT studies (i.e., observational, quasi-experimental), additional secondary outcomes in the psychosocial domain included anxiety, worry, and neuropsychiatric symptoms. More research is needed to examine these in more rigorous study designs. Neuropsychiatric symptoms which include anxiety, depression, as well as psychotic symptoms (e.g., delusions, hallucinations, agitation) and behavioral symptoms (e.g., disinhibition, aberrant motor behavior), are related to greater decline in global cognitive function in older adults with normal cognition or MCI [165] and specific cognitive domains such as memory XX, language, attention, and executive function [166] among patients with cognitive impairment. Identifying interventions that decrease neuropsychiatric or behavioral symptoms in older adults with MCI and AD/ADRD could decrease healthcare costs, as these symptoms often prompt earlier institutionalization, as they are difficult for caregivers to manage and increase caregiver burden [167].

### 4.2. Behavioral Mechanisms

Reported secondary outcomes from included RCTs under the behavioral domain include functional health and sleep. Sleep was the most reported secondary outcome for behavioral mechanisms in our included studies. We know that sleep appears to be associated with cognitive health. One study found that sleep quality was negatively associated with cognition and associated with a 5-year cognitive decline in U.S. adults [168]. Functional capacity, allowing aging individuals to maintain their activities of daily living and independence, is related to cognitive function [169]. Functional health appears to mediate the effects of age on global cognition and executive function in older adults [170]. Spiritual practices may support cognitive health by promoting functional health and sleep.

Additional secondary outcomes identified from non-RCT studies focused on associations with ADLs, which are used to assess a person’s daily functioning. These include daily functional tasks that an individual performs, such as dressing, bathing, toileting, etc. This builds on and mirrors findings above of functional health and balance reported in RCTs. Preserving functional task ability is incredibly important for older adults as it directly impacts their independence and autonomy, quality of life, and overall well-being.

### 4.3. Neurobiological Mechanisms

Mediating factors identified under the neurobiological domain included reduced inflammatory markers and improved immune response. Our findings suggest that spiritual practice that includes chanting, singing, breathing exercises, and repetitive poses, as seen in Kundalini yoga, may activate the parasympathetic nervous system, prompting reduced cortisol levels, promoting a state of relaxation, thus reducing stress and its inflammatory effects while supporting the function of the nervous system. This aligns with published research [116] reporting that religious and spiritual beliefs and values were associated with lower systemic inflammation (measured with CRP) over time in middle-aged and older adults using nationally representative population data. Elevated levels of inflammatory markers (e.g., CRP, interleukin-6 (IL-6), tumor necrosis factors) are related to cognitive decline and increased risk of AD/ADRD [171], found both in the peripheral blood and cerebral spinal fluid (CSF). A literature review and report found that chronic low-grade inflammation may impair cognitive performance, and peripheral inflammation induces neuroinflammation by disrupting the blood–brain barrier [172]. Immune response to infection with subsequent elevated inflammatory markers is also implicated in the amyloid β accumulation, a key pathological AD/ADRD factor [173]. Proper functioning of the body’s immune system supports the nervous system; thus, a disruption in the immune system may lead to impairment in cognitive function and neurogenesis [174]. Inflammation can disrupt the protective blood–brain barrier, imbalances in brain and immune system communication, and chronic inflammation contributing to neurodegeneration and loss of brain cells can lead to cognitive decline [175].

Further, recent literature reviews [176,177] report that mindfulness-based practices modulate the default mode network (DMN), which is implicated in mind wandering, attention, and emotional regulation. This reduced DMN activity may act as a mechanistic pathway influencing anxiety, depression, and cognitive decline; these neurocognitive shifts may reflect enhanced attentional control and cognitive flexibility, which are highly relevant to cognitive aging. More studies are needed to examine this potential mechanism for supporting better cognitive health in spiritual practice studies.

Secondary outcomes identified from non-RCT studies included improved fasting glucose levels and glycation end-products, telomerase activity, reduced stress, reduced inflammatory markers, increased synaptic plasticity, and improved reaction time in mental shifting; most reported were stress, inflammation, and synaptic plasticity. Diabetes is a risk factor for cognitive impairment, with research suggesting elevated levels of glycation end-products may link this association [178]. Thus, reducing these glycation end-products could support better cognitive function. Telomerase, an enzyme that is essential for extending and maintaining telomeres (the ends of chromosomes), is an important function in supporting telomere health, helping ameliorate the impact of toxic proteins (i.e., amyloid β) in neurogenerative diseases such as AD/ADRD [179]. Thus, practices that support telomerase activity in protecting telomeres may support better cognitive function and protect against AD/ADRD. Stress is a known risk factor for cognitive decline; thus, identifying practices to manage stress may reduce cognitive decline and support cognitive health [180]. Synaptic plasticity represents the brain’s ability to modify the strength of connections between neurons, a crucial process for maintaining brain health and function, supporting memory and learning [181]. Inducing and maximizing plasticity through spiritual practices can support better cognitive health. Through neurobiological pathways, spiritual practices may influence cognitive function through reduced inflammation, stress, regulating glucose and glycation end-product activity, while promoting more efficient brain function and synaptic health, and telomerase activity, supporting brain function and health.

Secondary outcomes that need additional testing were identified from included studies examining associations, including age-related gene-regulation expression and increased cerebral blood flow. DNA methylation is an epigenetic mechanism that plays a pivotal role in the pathogenesis of age-related neurodegeneration, such as AD/ADRD [182]; it regulates gene expression and influences neuronal development, synaptic plasticity, and the formation of memories, influencing brain and cognitive function [182,183]. Altering DNA methylation patterns may be a reversible mechanism influencing neurodegeneration, thus making it a modifiable way to support cognitive health and brain function. Cerebral blood flow is an essential element of cardiovascular health; it appears that reductions in cerebral blood flow are linked to cognitive decline [184] and may serve as a targetable mechanism to support better cognitive health.

#### 4.3.1. Additional Considerations

While most studies in this review conclude with positive outcomes, we note that one large-scale JAMA study found that among 585 older adults aged 65–84, the mindfulness intervention resulted in null findings [137]. These results highlight the heterogeneity across studies and suggest that benefits may vary by population, setting, or implementation. Recognition of this heterogeneity perhaps emphasizes the need for a more cautious interpretation and further investigation into the effectiveness of these spiritual practices.

#### 4.3.2. Implications

Several directions are possible regarding the more practical implications of this review. In terms of clinical practice, administering spiritual practices as a complementary or substitutional intervention could promote greater health among those who are experiencing or at risk for cognitive impairment. Adding spiritual activities to a structured lifestyle intervention, such as the POINTER study, could optimize cognitive health outcomes [185]. From a population perspective, public health departments can view the implementation of these spiritual practices as a preventative measure and as another strategic, supplemental intervention. These spiritual practices may be especially promising among sub-populations that may be wary of medication and/or other secular interventions. It is also essential to integrate the teaching of spiritual practice importance, particularly in fields of social science and ethics; training students and professionals to reflect on their own biases and cultivate an open-minded attitude toward spiritual practices may foster more inclusive cultural supports for aging adults [186]. As a culturally adaptable and low-cost intervention, both clinicians, public health officials, and instructors should seek to potentially implement these spiritual practices.

#### 4.3.3. Future Research

Findings from this review reveal the weakness of evidence on the causal mechanisms for underlying mechanisms. Future research is needed using longitudinal designs with repeated measures and explicit statistical mediation. The proposed model also needs to be tested. Included studies examined short- and medium-term effects of spiritual practices, but no studies examined long-term impacts of these practices. Determining if the long-term effects of spiritual practices could support dementia prevention or AD/ADRD modification is warranted. Additional studies examining over extended periods will provide a greater understanding of these mechanisms and spiritual practice effects. Future studies are also needed that explore spiritual practices across similar populations, as our included study populations varied across young and older adults, individuals with cancer, and older adults with mild cognitive impairment and mild dementia. Determining which spiritual practice may benefit which populations and if mechanisms differ across populations would provide a more targeted and personalized approach to supporting cognitive health.

We have presented our current findings, which appear to be mostly positive influence; however, emerging research also suggests that religion can act to exacerbate health outcomes. Therefore, future research should examine potentially negative aspects of religion as well [187].

#### 4.3.4. Strongest Evidence

The strongest evidence (RCTs) identified for potential mediators includes reduced systemic inflammatory markers and immune response through Kundalini yoga [133]. Secondary outcomes with the strongest (RCTs) evidence include perceived stress, depression, mental health, telomerase activity, sleep, mood, functional health, glucose, glycation end-products, and synaptic plasticity. It is important to note that intervention studies to establish causation in spiritual practices are extremely challenging and, in some cases, unethical. For example, extreme forms of spiritual practices such as uninterrupted prayer or deification, which require years of dedicated practice and are deeply ingrained in one’s lifestyle, may be challenging to examine. Scientists could create interventions around adults’ meaningful activities to support their continued involvement in practices that support cognitive health. This could look like a transportation intervention for older adults with cognitive impairment who may not be able to drive to their religious or spiritual service of choice. Many of these factors limit the possibilities for spiritual practice intervention studies, with meditation, mindfulness, tai chi, and yoga being more suitable for RCTs.

#### 4.3.5. Strengths and Limitations

The present review examined mediators that might help to explain the relationship between spiritual practices and cognitive health. Additional studies are needed to rigorously examine mediators identified in our review. We included cross-sectional studies that only reported secondary outcomes, with some studies using small sample sizes. Due to the vast heterogeneity of included study interventions and populations, the authors used a narrative approach to discuss findings that provide a base of general understanding of the current science regarding the underlying mechanisms of spiritual practice on cognitive health. This review serves as a starting point for future study of mechanisms across the different types of spiritual practices and may then provide meta-analysis opportunities for scientists to examine. Additional limitations include potential cultural or linguistic biases, including the exclusion of non-English studies, as included studies vary across continents and cultures. The meaning of spirituality may differ across cultures and populations, whereas some do not see yoga as a spiritual activity but more as an exercise.

These findings together offer a conceptual model to use as a framework for additional testing and in future studies to further examine mechanisms across the three domains described here. This work helps to fill the gap in the literature regarding how spiritual practices influence not only cognitive health, but mental and physical health more generally, thereby helping to inform the development of future interventions incorporating spiritual practices to improve cognitive health in later life.

## 5. Conclusions

Overall, in addition to affecting the main outcome of cognitive function, spiritual practices appear to also positively influence perceived stress, depressive symptoms, sleep quality, inflammatory markers, physiological markers of stress, and synaptic plasticity. The current evidence suggests that neurobiological factors account for the pathway between spiritual practice and cognitive health. Future research is needed using longitudinal designs with repeated measures and explicit statistical mediation. In addition, studies are needed that examine homogeneous interventions and populations to inform a greater understanding in this space. The proposed conceptual model also needs testing. Included studies examined short- and medium-term effects of spiritual practices, but no studies examined long-term impacts of these practices. We need to determine the long-term effects of spiritual practices and whether they could support AD/ADRD prevention and modification. Future investigations in these areas will further our understanding of how spiritual practices impact cognitive health. Identifying lifestyle factors such as spiritual practices that impact mediators known to increase AD/ADRD risk and adversely affect cognitive health will lead to interventions to optimize cognitive functioning with aging.

## Figures and Tables

**Table 1:** PubMed Search Terms and Strategy.

Databases	PubMed Using Official MeSH (Medical Subject Headings) and Keyword Terms
Search terms	(“Spirituality”[Mesh] OR “Spiritual Therapies”[Mesh] OR “Spiritualism”[Mesh] OR “Religion and Medicine”[Mesh] OR “Mind-Body Relations, Metaphysical”[Mesh] OR “Mind-Body Therapies”[Mesh] OR spiritual * OR religio *) AND (“Cognition”[Mesh] OR “Cognition Disorders”[Mesh] OR “Cognitive Dysfunction”[Mesh] OR “Cognitive Aging”[Mesh] OR “Social Cognition”[Mesh] OR “Memory Disorders”[Mesh] OR “Mental Fatigue”[Mesh]) OR “Memory”[Mesh] OR cognit * OR memory OR “mental health” OR depression OR anxiety OR hopeless OR energy OR appetite OR concentration OR restless OR nervous OR worry * OR irritable OR afraid) AND (Social Support”[Mesh] OR “Behavior and Behavior Mechanisms”[Mesh] OR “life purpose” OR meaning OR “Stress, Psychological”[Mesh] OR “Stress, Physiological”[Mesh] OR “psychological stress *” OR “physiological stress *” OR anxiety OR depression OR Inflamm * OR immune * OR cortisol OR sleep OR brain OR “brain structure” OR “brain function” OR “brain plasticity” OR neurogenesis OR MRI OR fMRI OR EEG OR ERPs OR (“diffusion sensor” AND imaging) OR BOLD OR VBM OR neurotransmitter OR BDNF OR biomarker OR neuroimmunology OR neurons OR glia OR vasculature OR IGF1 OR VEGF OR hormones OR peptides OR metabolism OR imaging OR neuromodulation OR volumetry OR neuromodulation OR endorphin OR monoamine OR dopamine OR noradrenaline OR norepinephrine OR serotonin OR opioid OR frontal lobes OR parietal lobes OR thalamus OR limbic OR cerebral blood) AND (spiritual *[tw]) AND ((2000/1/1:2023/12/31[pdat]) AND (english[Filter]) AND (alladult[Filter]))

* The asterisk indicates truncation, searching for all variations of the word root.

**Table 2:** Qualitative Study Ratings (n = 34).

Authors & Publication Year	Score Rating	Quality Rating
Meditation Studies (n = 13)		
Abomoelak et al., 2023	4/5	High
Bhattacharyya et al., 2023	4/5	High
Innes et al., 2018	5/5	High
Lavretsky et al., 2012	5/5	High
Lenze et al., 2014	5/5	High
Lenze et al., 2022	5/5	High
Oh et al., 2012	4/5	High
Meloni et al., 2013	5/5	High
Milbury et al., 2013	5/5	High
Mohan et al., 2011	5/5	High
Newberg et al., 2010	4/5	High
Singh et al., 2012	5/5	High
Spadaro & Hunker et al., 2016	4/5	High
Tai Chi Studies (n = 7)		
Chen et al., 2023	5/5	High
Hwang et al., 2020	5/5	High
Lam et al., 2012	4/5	High
Nguyen & Kraus, 2012	3/5	Medium
Port et al., 2018	4/5	High
Solianik et al., 2021	3/5	Medium
Sungkarat et al., 2018	4/5	High
Yoga Studies (n = 8)		
Eyre et al., 2017	4/5	High
Čekanauskaitė et al., 2020	3/5	Medium
Grzenda et al., 2024	3/5	Medium
Oken et al., 2006	5/5	High
Quigley et al., 2020	5/5	High
Rao et al., 2017	4/5	High
Sharma et al., 2005	3/5	Medium
Tremont, 2022	2/5	Low
General Spiritual and Religious Activity Studies (n = 6)		
Amir et al., 2022	4/5	High
Britt et al., 2023 [18]	4/5	High
Britt et al., 2023 [16]	4/5	High
Britt et al., 2024	4/5	High
Coin et al., 2010	4/5	High
Tai et al., 2015	1/5	Low

Note: The Mixed Methods Appraisal Tool (MMAT) was used to assess study quality and potential bias. Studies were categorized as high (≥80%), medium (60–80%), or low (≤40%) quality, based on the percentage of items addressed.

**Table 3:** Included meditation studies (n = 13).

Study Title	Objective	Authors (Year), Study Location	Study Population Total n (Mean Age +/− SD), % Female, % White; Group N’s; Group Demographics	Study Design; Time Points	Intervention; Comparison (If Relevant)	Domain: Potential Mechanism (Tool); Mediating Factor or Secondary Outcome	Cognitive Outcome Measure (Tool)	Effects on Outcomes (If Relevant)
Observational: longitudinal								
Protective roles of meditation practice and self-esteem on cognitive functions over time: Findings from the Midlife in the United States (MIDUS) study	Examine associations between meditation, self-esteem, and change over 10 years in midlife cognitive performance	Bhattacharyya et al., 2023 United States (US)	n = 2168 (mean age 64.7 +/− 11), 55.5% female, 90.8% White, 2.5% Black, 6.7% other race	Longitudinal Waves 2 & 3 of the MIDUS study	Relaxation /Meditation techniques in the past 12 months	Psychosocial Domain: Personality traits (agreeableness, conscientiousness, extraversion, openness, and neuroticism) (individual items) Secondary outcomes Psychosocial Domain: Self-esteem (7 items) mediating factor Neurobiological Domain: Self-reported physical health and chronic conditions secondary outcomes	Episodic verbal memory, working memory span and executive function, reasoning and speed of processing (Brief Test of Adult Cognition by Telephone (BTACT))	Persistent meditation practice at both waves was positively associated with episodic memory but not executive function. High self-esteem positively associated with both executive functions and episodic memory but did not mediate the association between meditation and cognitive performance.
Descriptive study								
Exploring the effects of an online asynchronous mindfulness meditation intervention with nursing students on stress, mood, and cognition: A descriptive study	Explore the effect of an online mindfulness meditation intervention with distance nursing students on stress, mood and cognition	Spadaro & Hunker et al., 2016 US	n = 26 (mean age not reported), 88% were Doctor of Nursing Practice (DNP) students, 81% White	A 24-week descriptive study 3 time points = baseline, 8 weeks, and 24 weeks ^ (^ only cognition was not measured at 24 weeks)	Mindfulness meditation 8-week online intervention (participants encouraged to practice during the week + write narrative response) plus additional practice at least 1 day per week (then posting narrative response)	Psychosocial Domain: Perceived stress (Perceived Stress Scale (PSS)), anxiety & depression (Hospital Anxiety and Depression Scale (HADS)) secondary outcomes	Attention: alerting, orienting, and executive control (Attention Network Test (ANT))	MBSR for online nursing students reduced stress after 8 weeks, maintained lower stress levels at 24 weeks. When practice frequency was weekly to daily, cognition: ability to shift attention, attention selection, concentration, and accuracy improved.
Quasi-Experimental								
Cognitive skills and DNA methylation are correlating in healthy and novice college students practicing Preksha Dhyāna meditation	Investigate the presence of any relationships in the changes in cognitive performance and DNA methylation in a college students practicing Preksha Dhyāna meditation	Abomoelak et al., 2023 US	n = 34 college students (mean age not reported), 82.4% female	Quasi-experimental 2 time points: baseline and 8 weeks	Preksha Dhyāna meditation 1 h total broken up over several sessions	Psychosocial Domain: Positive and negative aspect (Positive and Negative Affect Score (PANAS)) Neurobiological Domain: genetic testing (DNA methylation) secondary outcomes	Working memory (The Automated Working Memory Assessment—Short Form (AWMA-S)) Attention-related problems (The Conners Continuous Performance Test) Memory, attentional capacities (inattentiveness, impulsivity, and sustained attention)	Regular practice of Preksha Dhyāna meditation is associated with positive changes in cognitive skills and specific DNA methylation patterns among healthy college students. Sites of hypo- and hypermethylated patterns of cognitive skills after Preksha Dhyāna meditation were identified.
Mindfulness-Based Stress Reduction for older adults with worry symptoms and co-occurring cognitive dysfunction	Examine Mindfulness-Based Stress Reduction (MBSR) in older adults with worry symptoms and co-occurring cognitive dysfunction: (1) acceptability of MBSR, (2) whether MBSR needs to be lengthened providing more repetition, (3) MBSR’s benefits for worry reduction and cognitive improvements, and (4) continued use of MBSR techniques during follow-up	Lenze et al., 2014 US	n = 34 older adults worry and cognitive dysfunction (mean age not reported), 25% female, 29% White, 4% Black, 1% Hispanic	Quasi-experimental 3 time points: baseline, 3 months and 6 months	MBSR 8 sessions of MBRS vs. 12 sessions modified-MBRS	Psychosocial Domain: Mindfulness (Mindful Attention Awareness Scale (MAAS) & Cognitive Affective Mindfulness Scale-Revised (CAMS-R)), worry (Penn State Worry Questionnaire (PSWQ-A) Secondary outcomes	Immediate and delayed list and paragraph recall (Repeatable Battery for the Assessment of Neuropsychological Status (RBANS)) List recall (California Verbal Learning Test (CVLT))	MBSR reduced worry, increased mindfulness, and improved memory. No significant difference between 8-week and 12-week programs.
Preliminary evidence about the effects of meditation on interoceptive sensitivity and social cognition	Compare the effect of meditation practice on interoceptive sensitivity and related measures among long term practitioners (LTP), short term meditators (STM), and controls (nonmeditators)	Melloni et al., 2013 Argentina	n = 29 n = 10 LTP (mean practice years 2.17) (mean age 43.8 +/− 10.55; 30% female); n = 9 STM (mean practice weeks 8) (mean age 41.12 +/− 12.15; 44% female, n = 10 CG (mean age 37.30 +/− 9.12; 80% female)	Quasi-experimental Repeated measure between & within subjects	Meditation (compared LTP, STM, and CG)	Psychosocial Domain: Depression (Beck’s Depression Inventory (BDI)), anxiety (State Trait Anxiety Inventory (STAI)) Neurobiological Domain: Cardiac interoceptive sensitivity (Heartbeat detection task (HBD)) secondary outcomes	Mood state, executive function (INECO Frontal Screening (IFS) & social cognition tests)	Compared to CG, both meditators’ groups (STM and LTP) showed lower levels of anxiety and depression, but no improvement in executive function or social cognition performance.
Effects on cognitive function and cerebral blood flow in subjects with memory loss: A preliminary study	Determine if subjects with memory loss problems demonstrate changes in memory and cerebral blood flow after an 8-week meditation program	Newberg et al., 2010 US	n = 19 with memory loss n = 14 KK (mean age 64 +/− 8; 57% female). n = 5 Music group (mean age 65 +/− 10; 100% female)	Quasi-experimental 8-week meditation program vs. music listening, pre & post intervention	KK instructional video with demonstration followed by daily KK at home with CD music group: daily for 12 min each listen to violin music	Neurobiological Domain: changes in cerebral flood flow (CBF) (single photo emission computed tomography SPECT scans) secondary outcomes	Semantic memory (Category Fluency task) Cognitive function across attention, processing speed, executive function (Wechsler Adult Intelligence Scale (WAIS) Digit Symbol Substitution Test (DSST)) Verbal episodic memory (Logical Memory task) Psychomotor speed, visual search, attention (Trail Making Test Parts A and B)	The meditation program resulted in significant increases (*p* < 0.05) from baseline CBF ratios in the prefrontal, superior frontal, and superior parietal cortices with improvement on verbal fluency, TMT-B, and logical memory tests.
Immediate and long-term effects of meditation on acute stress reactivity, cognitive functions, and intelligence	The effects of meditation on stress-induced changes in physiological parameters, cognitive functions, intelligence, and emotional quotients	Singh et al., 2012 India	n = 34 young adults (mean age 24.4), 0% female	Quasi-experimental 2 phases: baseline & one month post treatment	Meditation Daily meditation × 1 month plus 15 min meditation after playing a stressful computer game	Psychosocial Domain: Perceived stress (Cohen Perceived Stress Scale) Neurobiological Domain: Stress (Computerized game stressor, salivary cortisol, Stanford Acute Stress Reaction Questionnaire (SASRQ)), Cardiac health (Heart rate), electrical conductivity of the skin (Galvanic Skin Response (GSR) secondary outcomes	Working memory (Sternberg memory test (MEMSCAN)), cognitive flexibility (Stroop color interference test) reasoning ability (Intelligent quotient test)	Meditation reduced psychological stress responses (GSR and AS) and enhanced cognitive functions; benefits were more pronounced after one month of consistent practice.
Randomized Control Trials (RCTs)								
Effects of meditation and music-listening on blood biomarkers of cellular aging and Alzheimer’s disease in adults with subjective cognitive decline: An exploratory randomized clinical trial	To assess the: (1) effects of two 12-week relaxation programs on telomere length (TL), telomerase activity (TA), and plasma amyloid-β (Aβ) levels in adults with subjective cognitive decline; and (2) relationship between biomarker changes and cognitive function, psychosocial status, and quality of life (QOL)	Innes et al., 2018 US	n = 53 middle-age to older adults with subjective cognitive decline (mean age 60.5), 87% female, 94% non-Hispanic White	12-week RCT Baseline, 3 months, 6 months	Kirtan Kriya meditation (KK) or music listening (ML) KK used a CD for 12 min daily meditation sessions ML 12 min daily	Neurobiological Domain: peripheral blood mononuclear cell of Telomere length (TL), cell proliferation (telomerase activity (TA)), plasma amyloid-β(Aβ) Psychosocial Domain: mood (65-item Profile of Mood States), well-being (Psychological Well-Being Scale, perceived stress (Perceived Stress Scale), health-related QOL (36-item MOS Short Form) Behavioral Domain: sleep quality (Pittsburgh Sleep Quality Index) secondary outcomes	Memory function (Memory Functioning Questionnaire (MFQ)), executive function (Trail Making Test Parts A and B), working memory (the 90 s Wechsler Digit-Symbol Substitution Test (DSST))	Both groups improved in cognitive and psychosocial status (*p* ≤ 0.05), with improvements in stress, mood, and QOL greater in the KK group. Rising Aβ levels were associated with gains in cognitive function, mood, sleep, and QOL at both 3 and 6 months.
A pilot study of yogic meditation for family dementia caregivers with depressive symptoms: Effects on mental health, cognition, and telomerase activity	To examine the effects of daily yogic meditation on mental health, cognitive functioning, and immune cell TA in family dementia caregivers with mild depressive symptoms	Lavretsky et al., 2012 US	n = 39 family dementia caregivers n = 23 KK group (mean age 60.5 +/− 28.2; 100% female); n = 16 relaxation group (mean age 60.6 12.5; 87.5% female)	RCT baseline and 8 weeks	Kirtan Kriya meditation 12 min daily	Psychosocial Domain: Depressive symptoms (Hamilton Depression Rating Scale (HAM-D), mental and physical health-related QOL (SF-36), global chronic medical illness burden (Cumulative Illness Rating Scale) Neurobiological Domain: Cell proliferation (TA) secondary outcomes	Cognition (Mini-mental State Examination (MMSE)) Verbal memory (California Verbal Learning Test-II (CVLT II)) attention & speeded information processing (TMT-A) executive function (TMT-B)	M group had significantly fewer depressive symptoms and greater improvement in mental health (*p* < 0.05), cognitive and executive function (*p* < 0.05), and improvement in TA (*p* = 0.05) vs. the relaxation group.
Effects of mindfulness training and exercise on cognitive function in older adults: A randomized clinical trial	To determine whether MBSR, exercise, or a combination of both improve cognitive function in older adults	Lenze et al., 2022 US	n = 585 older adults (mean age 71.5 +/− 4.8), 72.5% female, 81.5% non-Hispanic White, 11.8% non-Hispanic Black, 4.6% Asian, 0.3% American Indian, 1.8% unknown or >1 race. 6.7% Hispanic n = 150 MBSR Exercise group n = 138 Combined MBSR + exercise n = 144 CG n = 153	RC 3 time points: baseline, 6 months & 18 months	MBSR, exercise, combined MBSR + exercise, CG 18 months intervention: MBSR 1 h daily; exercise 300 min weekly; combined MBSR and exercise; health education CG with monthly in-person classes	Psychosocial Domain: QOL (Quality of Life in Neurological Disorders Cognitive Function), mindfulness state (Cognitive and Affective Mindfulness Scale–Revised (CAMS-R), sleep (time to fall asleep and total sleep time) Behavioral Domain: physical performance (accelerometer, treadmill performance testing), activities of daily living (Revised Observed Tasks of Daily Living) Neurobiological Domain: aerobic fitness, insulin sensitivity & resistance, body fat and fat-free masses, stress (plasma cortisol levels), upper- and lower-body strength (bi-weekly classes & self-reported surveys), Hippocampal volume, dorsolateral prefrontal cortex thickness, surface area (MRI) secondary outcomes and mediating factors	Memory (immediate and delayed recall using a 16-item word list, the Picture Sequence Memory Test) Executive function (Dimensional Change Card Sort test, Flanker Inhibitory Control and Attention Test, List Sorting Working Memory Test, Consonant-Vowel Odd-Even Switching test, the Sustained Attention to Response Test, and the Stroop Test).	Among older adults with subjective cognitive concerns, mindfulness training, exercise, or both did not result in significant differences in improvement in episodic memory or executive function scores at 6 months. No significant improvements due to the interventions at 18 months in secondary outcomes, including structural brain measures of hippocampus and dorsolateral prefrontal cortex (DLPFC).
Tibetan sound meditation for cognitive dysfunction: Results of a randomized controlled pilot trial	Investigate the role of mind–body programs in the cognitive rehabilitation of breast cancer survivors	Milbury et al., 2013 USA	n = 37 cognitively impaired breast cancer survivors (mean age 56.3), 100% female, 59.5% White, 5.4% African American, 10.8% Asian American, 24.3% Latino-Hispanic	RCT 3 time points: baseline, end of treatment (6 weeks) and 1-month post-treatment	Tibetan Sound meditation (TSM) program vs. wait list CG 2 weekly TSM sessions for 6 weeks; CG received usual care and were asked to refrain from mediation participation	Psychosocial Domain: Health-related QOL (SF-36), depressive symptoms (Center for Epidemiologic Studies Depression Scale), spiritual well-being (Functional Assessment of Chronic Illness Therapy—Spiritual Well-being scale (FACIT-Sp)) Behavioral Domain: sleep disturbances (Pittsburgh Sleep Quality Index), fatigue (Brief Fatigue Inventory) secondary outcomes	Cognitive function (Functional Assessment of Cancer Therapy (FACT)-Cog), verbal memory (Rey Auditory Verbal Learning Test), short-term memory and processing speed (Digit Symbol)	Compared to CG, women in the TSM group performed better on the verbal memory test (*p* = 0.06) and the short-term memory and processing speed task (*p* = 0.09) and reported improved cognitive function (*p* = 0.06), cognitive abilities (*p* = 0.08), mental health (*p* = 0.04), and spirituality (*p* = 0.05) at the end of treatment but not 1 month later. Women in TSM group had significantly fewer depressive symptoms (*p* = 0.05), better mental health (SF-36) (*p* < 0.04), and spiritual well-being (*p* = 0.05).
Effect of meditation on stress-induced changes in cognitive functions	To examine the effects of meditation on stress-induced changes in cognitive function	Mohan et al., 2011 India	n = 32 young adults with no history of meditation (mean age 27.3 +/− 1.8), 0% females	RCT pre and post intervention	Meditation, CG 2 interventions (stressful computer game preceded & followed by meditation) & 2 control groups (20 min wait time)	Neurobiological Domain: Electrical activity of muscles (electromyography (EMG)), electrical activity of the heart (electrocardiography (ECG)), skin’s electrical conductivity changes in response to emotional stimuli (galvanic skin response (GSR)), heart health (phonocardiography recordings), sympathetic reactivity (QTc/QS2 ratio), stress (cortisol & acute psychologic stress scores) secondary outcomes	Memory (Wechsler memory scale and visual-choice reaction time (VCRT)	Computer game stress led to significant increases in both physiological (GSR, EMG, HR, QTc/QS2) and psychological (acute stress scores) stress markers. Meditation promotes relaxation. When practiced before a stressful event, meditation mitigated the adverse effects of stress. Memory quotient improved, and cortisol levels decreased following both stress and meditation. No significant change observed in VCRT.
Effect of medical Qigong on cognitive function, quality of life, and a biomarker of inflammation in cancer patients: A randomized controlled trial	Evaluates the effects of medical Qigong (MQ); combination of gentle exercise and meditation) on cognitive function, QOL, and inflammation in cancer patients	Oh et al., 2012 Australia	n = 81 cancer patients (mean age 62) MQ n = 37 CG n = 44	Stratified RCT 2 time points: baseline and 10 weeks medical Qigong vs. usual care	MQ, CG MG 90 min group sessions weekly × 10 weeks; CG received usual care	Psychosocial Domain: Quality of life (Functional Assessment of Cancer Therapy—General (FACT-G) systemic inflammation (C-reactive protein (CRP)) secondary outcomes	Cognitive function (FACT-Cog)	MQ group self-reported significantly improved perceived cognitive impairment, perceived cognitive impairment on QOL, and perceived cognitive abilities compared to controls (*p* < 0.05). The MQ group reported significantly improved QOL, had reduced CRP levels compared to controls (*p* < 0.05).

Notes: If some study details, such as race, ethnicity, mean age, standard deviation, etc., are not listed, it is because studies did not report that information. Abbreviations: Aβ = plasma amyloid-β; ANT = Attention Network Test; AWMA-S = The Automated Working Memory Assessment—Short Form; BDI = Beck’s Depression Inventory; BTACT = Brief Test of Adult Cognition by Telephone; CAMS-R = Cognitive and Affective Mindfulness Scale–Revised; CBF = cerebral flood flow; CG = Control Group; CRP = C-reactive protein; CVLT = California Verbal Learning Test; CVLT II = California Verbal Learning Test-II; DNP = Doctor of Nursing Practice; DSST = Digit Symbol Substitution Test; ECG = electrocardiography; EMG = electromyography; FACIT-Sp = Functional Assessment of Chronic Illness Therapy—Spiritual Well-being scale; FACT-Cog = Functional Assessment of Cancer Therapy-Cognitive Function; GSR = galvanic skin response; HADS = Hospital Anxiety and Depression Scale; INECO–IFS = Institute of Cognitive Neurology Frontal Screening; LTP = Long term practitioners; MAAS = Mindful Attention Awareness Scale; MBSR = Mindfulness-Based Stress Reduction; MFQ = Memory Functioning Questionnaire; MIDUS = Midlife in the United States; MQ = medical Qigong; PANAS = Positive and Negative Affect Score; PSS = Perceived Stress Scale; PSWQ-A = Penn State Worry Questionnaire; QOL = quality of life; RBANS = Repeatable Battery for the Assessment of Neuropsychological Status; SASRQ = Stanford Acute Stress Reaction Questionnaire; STAI = State Trait Anxiety Inventory; STM = short term meditators; TL = telomere length; TA = telomerase activity; TSM = Tibetan Sound meditation; US = United States; WAIS = Wechsler Adult Intelligence Scale.

**Table 4:** Included Yoga studies (n = 8).

Study Title	Objective	Authors (Year), Study Location	Study Population (n) (Mean Age +/− SD), % Female, % White; Group (Final n), Demographics	Study Design; Time Points	Intervention; Comparison (If Relevant)	Domain: Potential Mechanism (Tool); Mediating Factor or Secondary Outcome	Cognitive Outcome Measure (Tool)	Effects on Outcomes (If Relevant)
Randomized Control Trials (RCTs)								
A 10-week yoga practice has no effect on cognition but improves balance and motor learning by attenuating brain-derived neurotrophic factor levels in older adults	Investigate the effects of yoga on cognition, balance under single- and dual-task conditions, and motor learning	Čekanauskaitė et al., 2020 Lithuania	n = 33 older adults n = 18 Yoga N = 15 Control (mean age 66.9 +/− 6.0) 91% female	RCT 2 time points: Baseline & 10 weeks	Yoga vs. Control 2 90 min yoga session per week × 10 weeks (20 sessions total) Control group maintained daily living habits	Psychosocial Domain: Depression (Brunel Mood Scale) Secondary outcome	Mental flexibility, verbal working memory, response inhibition, and visuospatial processing (Automated Neuropsychological Assessment Metrics).	No statistical significance between yoga and cognitive measures or depression.
A randomized controlled trial of Kundalini yoga in mild cognitive impairment	To investigate the effects of Kundalini yoga training compared to Memory enhancement training (MET) on MCI	Eyre et al., 2017 US	n = 79 mild cognitive impairment n = 38 KY (mean age 68.1 +/− 8.7, 65.8% female, 63.2% White) n = 41 MET (mean age 67.6 +/− 8.0; 65.9% female, 73.2% White)	RCT 3 time points: baseline, 12 weeks, & 24 weeks	KY vs. MET 60 min KY weekly, & daily KY × 12 min for 12 weeks 20 min daily MET	Psychosocial Domain: Depression (GDS) Secondary outcome	Verbal memory (Hopkins Verbal Learning Test (HVLT) & Wechsler Memory Scale (WMS-IV)); visual-spatial skills & visual memory (Rey Osterrieth (Rey-O)); executive function (Trail Making Test (TMT), Stroop Word-Color Test, and Animal Naming Test)	At 12 weeks and 24 weeks, both KY and MET groups showed significant improvement in memory; however, only KY showed significant improvement in executive functioning. Only the KY group showed significant improvement in depressive symptoms and resilience at week 12.
Cognitive and immunological effects of yoga compared to memory training in older women at risk for Alzheimer’s disease	Assess the efficacy of Kundalini yoga training (KY) compared to memory enhancement training (MET) on mood and cognitive functioning in a group of older women with CVRFs and SCD	Grzenda et al., 2024 US	n = 79 older women at risk of AD n = 40 KY (mean age 65.45 +/− 9.11, 100% female, 68% White), n = 39 MET (mean age 67.54 +/− 9.30, 100% female, 64% White)	RCT 2 time points: 12 weeks & 24 weeks	KY vs. MET 24 weeks KY 1 h weekly in-person lesson × 12 weeks plus 12 min daily home exercise MET weekly in-person class × 12 weeks plus 20 min daily exercise	Psychosocial domains: Perceived stress (PSS), health related QOL (SF-36) Behavioral Domain: Depression (BDI), anxiety (HAM-A), resilience (CD-RISC-25) Neurobiological Domain: systemic inflammation (cytokines & chemokines) Mediating factor	Delayed recall (Hopkins Verbal Learning Test- revised, Wechsler memory Scale-IV, & Re-Osterreith Complex Figure); executive function (Stroop Interference & TMT-B); subjective memory (MFQ)	At 24-week follow-up, KY yielded a significant, large effect size improvement in subjective cognitive impairment measures compared to MET. KY on a transcriptional level, at 12- and 24-week follow-up, KY uniquely altered aging-associated signatures, including interferon gamma and other psycho-neuro-immune pathways. Levels of chemokine eotaxin-1, an aging marker, increased over time in MET but not KY participants.
Randomized, controlled, six-month trial of yoga in healthy seniors: Effects on cognition and quality of life	To determine the effect of yoga on cognitive function, fatigue, mood, & QOL in older adults	Oken et al., 2006 US	n = 135 healthy senior people n = 47 Walking exercise group (mean age 73.6 +/− 5.1, 78.7% female, 76.6% White) n = 44 Yoga (mean age 71.5 +/− 4.9, 70.4% female, 95.5% White) n = 44 Wait list (mean age 71.2 +/− 4.4, 75.0% female, 86.4% White)	RCT 2 time points: baseline & at 6 months	Hatha Yoga vs. Walking Exercise Class vs. Wait list 1 class per week × 6 months: Hatha yoga, walking exercise class, or wait-list control	Psychosocial Domain: Depression (CESD) Behavioral Domain: fatigue (POMS) secondary outcomes	Alertness, attention (Stroop Color and Word Test), quantitative electroencephalogram (EEG)	There were no effects from either of the active interventions on any of the cognitive and alertness outcome measures or depression measures.
Feasibility and impact of a yoga intervention on cognition, physical function, physical activity, and affective outcomes among people living with HIV: A randomized controlled pilot trial	Assess the feasibility and impact of a triweekly 12-week yoga intervention among people living with HIV	Quigley et al., 2020 Canada	n = 22 adults with HIV (mean age 55.5 +/− 10.7, 22.7% female, 72.7% White) n = 11 Yoga (mean age 50.7 +/− 10.2, 9.1% female, 81.8% White) n = 11 CG (mean age 60.2 +/− 9.2, 36.4% female, 63.6% White)	RCT 2 time points: Baseline & 12th week	Yoga vs. control 12-week 60 min yoga intervention CG maintained usual physical activity levels at home	Psychosocial Domain: Depression (HADS) Secondary outcome	Cognitive performance (Brief Cognitive Ability Measure (B-CAM) & Communicating Cognitive Concerns Questionnaire (C3Q)).	Intention-to-treat analyses showed no significant within- or between-group differences in cognitive and physical function. In contrast with the intention-to-treat analyses, per protocol analyses showed a trend in improved cognitive subscale scores and no trend in improved health transition scores among yoga participants. There were also difference trends among yoga participants on the HADS–Depression subscales.
Effects of mind sound resonance technique (yogic relaxation) on psychological states, sleep quality, and cognitive functions in female teachers: A randomized, controlled trial	To examine the effects of a mind sound resonance technique (MSRT) intervention on perceived stress, quality of sleep, cognitive function, state and trait anxiety, psychological distress, and fatigue among female teachers	Rao et al., 2017 India	n = 60 adult female teachers n = 30 MSRT (mean age 43.0 +/− 9.77, 100% female) n = 30 CG (mean age 40.0 +/− 7.32, 100% female)	RCT 2 time points: Baseline & 1 month	MSRT vs. CG MSRT 5 days per week × 1 month CG (normal daily routines)	Behavioral Domain: Sleep (PSQI), Anxiety (Spielberg’s State-Trait Anxiety Inventory), Psychological Distress (GHQ-12), self-esteem (Rosenberg’s self-esteem scale), perceived stress (Cohen’s perceived stress scale (PSS)) Behavioral Domain: fatigue (Revised version Piper Fatigue Scale (PFS)) Secondary outcomes	Scanning memory, psychomotor speed, and visual attention (Digit Letter Substitution Test)	There were no significant cognitive differences between MSRT and CG (*p* = 0.083). Importantly, cognitive function declined in the CG by the end of the month (*p* = 0.002). In the MSRT group, significant decreases were seen in perceived stress (*p* < 0.001), state anxiety (*p* < 0.001), trait anxiety (*p* < 0.001), psychological distress (*p* < 0.001), and fatigue (*p* = 0.005), together with significant improvements in sleep quality (*p* < 0.001) and self-esteem (*p* < 0.001).
Effect of Sahaj Yoga on neuro-cognitive functions in patients suffering from major depression	Observe the effect of yoga practice on cognitive functions among the patients of depression	Sharma et al., 2005 India	n = 30 adults with major depression n = 15 Yoga (mean age 31.87 +/− 8.78, 33% female) n = 15 CG (mean age 31.67 +/− 8.46, 40% female)	RCT 2 time points: Baseline & 8th weeks	Sahaj Yoga vs. control 8-week 30 min 3× per week Sahaj Yoga CG (anti-depressant medication)	Psychosocial Domain: depression (HAM-D) Secondary outcome	Attention span, visuo-motor speed (Letter Cancelation Test), short-term memory, working memory (Digit Span), and executive functions (TMT-A & B, & Ruff Figural Fluency Test)	Table II demonstrates percentage reduction in HAM-D scores at 8 weeks was significantly more in Yoga patients than in CG (*p* = 0.003). Also, there is more significant change in number of Omissions and Letter cancelation time in Yoga subjects as compared to CG. Significant improvement is seen in TMT-A & TMT-B’ in both the groups with no inter-group differences.
Feasibility of a yoga intervention for individuals with mild cognitive impairment: A randomized controlled trial	To examine the effect of a yoga intervention for individuals with MCI vs. healthy living education classes (HLE) on neuropsychological and mood measures	Tremont 2022 US	n = 46 older adults with MCI n = 25 Yoga (mean age 71.56 +/− 5.80, 60% female, 100% White) n = 21 HLE (mean age 71.67 +/− 6.27, 7% female, 100% White)	RCT 2 time points: baseline, & 12 weeks	Yoga intervention (YI) vs. HLE Classes 2× 1 h yoga classes per week, 2× 1-h HLE classes per week	Psychosocial Domain: Depression (CES-D) Secondary outcome	Attention (TMT-A, Digit Span Forward), executive function (Letter Fluency, TMT-B, Digit Span Backward, Letter–Number Sequencing), language (semantic fluency), memory (Hopkins Verbal Learning Test–Revised, Brief Visuospatial Memory Test–Revised), visual spatial (Rey–Osterrieth Complex Figure).	Participants in both conditions reported high levels of satisfaction and reasonable class attendance rates. Home practice rates were low. There were no adverse events deemed related to the YI. Results showed a medium effect size in favor of the YI in visuospatial skills. The yoga group also showed a large effect size indicating decline in perceived stress compared with the HLE group, whereas HLE resulted in greater reductions in depressive symptoms after the intervention (large effect size).

Notes: If some study details, such as race, ethnicity, mean age, standard deviation, etc., are not listed, it is because studies did not report that information. Abbreviations: AD = Alzheimer’s disease; BDI = Beck Depression Inventory; CD-RISC-25 = Connor Davidson Resilience Scale; CESD = Center for Epidemiologic Studies Depression Scale; CG = Control group; CVRFs = Cerebrovascular Risk Factors; GDS = Geriatric Depression Scale; GHQ-12 = General Health Questionnaire-12; HADS = Hospital Anxiety and Depression Scale; HAM-D = Hamilton Depression Rating scale; HAM-A = Hamilton Anxiety Rating Scale; HLE = healthy living education classes; HVLT = Hopkins Verbal Learning Test; KY = Kundalini yoga training; MET = Memory enhancement training; MFQ = Mood and Feelings Questionnaire; MSRT = Mind Sound Resonance Technique; P PFS = Piper Fatigue Scale, Revised version; POMS = Profile of Mood States; Decline; PSS = Cohen’s Perceived Stress Scale; PSQI = Pittsburgh Sleep Quality Index; QOL = quality of life; Rey-O = Rey Osterrieth test; SCD = Subjective Cognitive; SF-36 = Short Form Survey; TMT-A = Trail Making Test—Part A; TMT-B = Trail Making Test—Part B; WMS-IV = Wechsler Memory Scale, Version 4; YI = Yoga intervention.

**Table 5:** Included Tai Chi studies (n = 7).

Study Title	Objective	Authors (Year), Study Location	Study Population (n) (Mean Age +/− SD), % Female, % White; Group (Final n), Demographics	Study Design; Time Points	Intervention; Comparison (If Relevant)	Domain: Potential Mechanism (Tool); Mediating Factor or Secondary Outcome	Cognitive Outcome Measure (Tool)	Effects on Outcomes (If Relevant)
Observational Studies: Cross-sectional studies								
Cognition and brain function in elderly Tai Chi practitioners: A case-control study	To compare cognition and brain function between Tai Chi and Water Aerobic older adult practitioners.	Port et al., 2018 Brazil	Older adults without severe cognitive impairment: n = 8 TC (mean age 66.4 +/− 4.9, 62.5% female) n = 8 WA (mean age 66.4 +/− 7.0, 62.5% female)	cross-sectional, case–control, baseline	TC vs. WA: required history of at least 3 years TC or WA practice, two times weekly Inclusion criteria required at least 3 years of practice in either TC or WA	Psychosocial Domain: Depression (Beck Depression Inventory), anxiety (Beck Anxiety Inventory), mental health (Self-reporting questionnaire) Secondary outcomes Neurobiological Domain: brain region activation/response during attention & working memory testing (fMRI scans) Potential mediating factor	Attention and working memory (Digit span test); visuomotor coordination, attention and processing speed (Digit symbol test); verbal fluency (Controlled oral word association test); attention and processing speed (Trail Making Test-Part A (TMT-A)); attention and executive function (Trail Making Test—Part B (TMT-B)); verbal memory (Rey Auditory Verbal Learning Test (RAVLT)); attention and cognitive flexibility (Stroop Word-Color Task), working memory capacity and function (N-back tasks)	No significance in cognitive tests between TC and WA groups; TC had lower anxiety (*p* < 0.020) than WA; TC had smaller brain activation in 3 areas (right intracalcarine cortex, lateral occipital cortex, and occipital pole) during Stroop Word Color Task and in 2 areas (right frontal pole and superior frontal gyrus) during N-back testing vs. WA.
Randomized Control Trials (RCTs)								
Effects of Tai Chi Chuan on cognitive function in adults 60 years or older with type 2 Diabetes and mild cognitive impairment in China: A randomized clinical trial	Compare the effectiveness of Tai Chi Chuan with T2D and MCI, with fitness walking.	Chen et al., 2023 China	n = 328 MCI with T2D n = 107 TC (mean age 67.56 +/− 4.99, 54.2% female) n = 10 Walking Group (WG) (mean age 67.46 +/− 4.73, 44.5% female). n = 11 control (mean age 67.62 +/− 5.35, 54.1% female)	RCT 3 time points: baseline, 24 weeks, & 36 weeks	TC, WG, and control had education seminars once every 4 weeks × 24 weeks; TC: supervised 24-week, 24-form TC training—1 h session 3 × weekly involving MB exercise, synchronous breathing, & movement patterns; WG: supervised 24-week fitness walking program—1 h 3 × weekly involving walking; TC & WG encouraged to continue on own after 24 weeks; control group: no exercise intervention, maintained previous lifestyle	Neurobiological Domain: Metabolic indices (fasting glucose, insulin resistance (HOMA-IR), glycated hemoglobin (HbA1C), advanced glycation end-products & their receptors (ratio of advanced glycation end products (AGE), soluble receptor of AGE (sRAGE)) secondary outcomes and potential mediating factors	Global Cognition (MoCA); cognitive subdomains: memory (Wechsler Memory Quotient (MQ), attention/processing speed/executive function (Digit Symbol Substitution Test (DSST)), executive control (TMT-B), word retrieval ability (Boston Naming Test (BNT)), visuo-constructional ability/visual memory (Rey-Osterrieth Complex Figure Test (ROCF)).	TC [vs. WG & CG] improved memory (*p* < 0.001) and fasting glucose levels (*p* < 0.05); TC [vs. WG only] also improved Global Cognition (*p* < 0.001) and advanced glycation end-products and their receptors (*p* < 0.001); TC [vs. CG only] also improved attention/processing speed/executive function (*p* < 0.05) and executive control (*p* < 0.05). No significant differences between groups in other outcomes or potential mediating factors (BNT and ROCF scores, HbA1c level, and HOMA-IR) between TG and WG or CG.
Effects of computerized cognitive training and tai chi on cognitive performance in older adults with traumatic brain injury	Compare the effects of computerized cognitive training (CCT) and tai chi (TC) with usual care (control) on cognitive functions and secondary outcomes in older adults with traumatic brain injury.	Hwang et al., 2020 Taiwan	n = 96 older adults with cognitive impairment with traumatic brain injury n = 32 CCT (mean age 65.8 +/− 10.7, 46.9% female) n = 32 TC (mean age 65.8 +/− 9.9, 56.2% females) n = 32 control (mean age 68.1 +/− 11.4, 62.5% females)	RCT 3 time points: baseline, 6 months, 12 months	CCT included software program of 4-domain cognitive training (i.e., attention, memory, speed of processing, executive function) with study nurse at residence for 45 min once a week × 6 months plus participants asked to self-practice 3 × per week; TC was Yang-style with instructor at residence for 50 min once per week × 6 months plus participants instructed to practice 3 times per week on own; and control group had study nurse visits every 2 weeks × 6 months & monthly telephone contacts	Psychosocial Domain: Depression (CESD) Behavioral Domain: Handgrip strength (handgrip dynamometer), lower extremity strength (5 sit-to-stands), balance (Tinetti balance test), independence (Activities of Daily Living (ADLs), disability (Extended Glasgow Outcome Scale (GOSE)) secondary outcomes	Cognitive function (Mattis Dementia Rating Scale (MDSR), Mini-Mental State Examination (MMSE), modified Telephone Interview of Cognitive Status (TICS-M), TMT-B.	TC [vs CG] increased cognition scores (MDRS total by 4.39 (ES = 0.54) points and conceptualization by 1.94 (ES = 0.59) points, MMSE by 1.75 (ES = 0.80) points) at 6 months and cognition (MDRS total by 4.14 (ES = 0.39) points and initiation/preservation by 2.04 (ES = 0.26) points, TICS-M by 3.04 (ES = 0.58) points, reduced time to complete TMT-B by 23.1 (ES = 0.59) seconds at 12 months; reduced time to complete 5 sit-to-stands (lower extremity strength) by 3.20 (ES = 0.50) seconds at 6 months. Only TC [vs CCT] had 12-month significant findings on cognition. No significance in other secondary outcomes.
A 1-Year randomized controlled trial comparing mind body exercise (Tai Chi) with stretching and toning exercise on cognitive function in older Chinese adults at risk of cognitive decline	compare the effectiveness of Chinese-style mind–body exercise (24 forms simplified Tai Chi) versus stretching and toning exercise in the maintenance of cognitive abilities in Chinese elders at risk of cognitive decline.	Lam et al., 2012 Hong Kong	n = 389 older adults with MCI n = 171 TC (mean age 77.2 +/− 6.3, 73% female) n = 218 control (mean age 78.3 +/− 6.6, 79% female)	RCT 4 time points: Baseline, 5, 9, & 12 months.	TC had training on 24 simplified Tai Chi forms; control group practiced muscle-stretching and toning exercises; both TC and control group had instructors conduct weekly sessions at training centers × 4–6 weeks followed by participants practicing at home with a video CD for 30 min per day, 3 days per week	Psychosocial Domain: Depression (CSDD), neuropsychiatric symptoms (Chinese NPI). Behavioral Domain: functional balance (BBS) secondary outcomes	Dementia progression (DSM-IV rate), cognition (ADAS-Cog), digit span, delay recall, category verbal fluency tests, TMT, MMSE).	TC vs. CG had lower risk of developing dementia (*p* = 0.04), better preservation of cognition (CDR scores) (*p* = 0.004), better functional balance (*p* = 0.02), and greater improvement in delayed recall (*p* = 0.05) and depression in dementia scores (*p* = 0.02) at 12 months. No significance in NPI, MMSE, ADAS-Cog, digit span, category verbal fluency tests, or TMT.
A randomized controlled trial of Tai Chi for balance, sleep quality and cognitive performance in elderly Vietnamese	Evaluate the effects of TC on balance, sleep quality, and cognitive performance in community-dwelling older adults	Nguyen & Kraus, 2012 Vietnam	n = 102 community-dwelling older adults n = 48 TC (mean age 69.23 +/− 5.3, 50% female) n = 48 control (mean age 68.73 +/− 4.95, 50% female)	RCT 2 time points: baseline & 6 months	TC was 24-form for 60 min training 2 × s per week × 6 months; control group maintained normal activities	Behavioral Domain: Balance (Falls Efficacy Scale (FES), sleep (Pittsburgh Sleep Quality Index (PSQI) secondary outcomes	Motor speed and visual attention (TMT_A &B).	TC vs. control group had greater improvement in cognition (TMT-A (*p* < 0.001) and TMT-B (*p* < 0.001)), better scores in balance ability (*p* < 0.001) and sleep (*p* = 0.001).
Tai Chi improves psychoemotional state, cognition, and motor learning in older adults during the COVID-19 pandemic	Determine the effect of a 10-week tai chi intervention on psychoemotional state, cognition, and motor learning in older adults during the COVID-19 pandemic	Solianik et al., 2021 Lithuania	n = 30 older adults n = 15 TC (86.67% female) n = 15 CG (86.67% female)	RCT 2 time points: baseline & 10 weeks	TC was 8-form, Yang-style, biweekly 60 min classes per week × 10 weeks with instructor; control group maintained daily routines	Psychosocial Domain: Anxiety and depression symptoms (HADS), perceived stress (PSS-10) Behavioral Domain: Motor performance (DPA-1) Neurobiological Domain: Synaptic plasticity (BDNF), physiological stress (HR and HR variability, BP) secondary outcomes	Cognitive performance (ANAM-4) with visuospatial processing (MGT), verbal working memory (MST), response inhibition (GNGT), and choice reaction time and ability to shift mental set (PRTT).	TC increased synaptic plasticity (*p* < 0.001, CE = 1.39) decreased perceived stress (*p* < 0.05, CE = 0.83), decreased depression (*p* = 0.034, CE = 0.62), improved reaction time for motor performance (*p* < 0.037, CE > 0.87), improved reaction time in mental shifting (*p* = 0.014, CE = 0.87), improved accuracy in visuospatial processing (*p* = 0.017, CE = 0.79), and improved accuracy in response inhibition (*p* = 0.005, CE = 0.27); TC vs. CG decreased perceived stress (*p* < 0.001), greater accuracy in visuospatial processing (*p* = 0.012). No significance in physiological stress.
Tai Chi improves cognition and plasma BDNF in older adults with mild cognitive impairment: A randomized controlled trial	Examine the effects of TC on cognitive functions and plasma biomarkers (brain-derived neurotrophic factor [BDNF], tumor necrosis factor-α [TNF-α], and interleukin-10 [IL-10]) in a-MCI.	Sungkarat et al., 2018 Thailand	n = 66 a-MCI n = 33 TC (mean age 68.3 +/− 6.7, 93.9% female) n = 33 CG (mean age 67.5 +/− 7.3, 78.8% female)	RCT 2 time points: Baseline & 6 months	TC 10-form learned from instructor for 9 sessions (3 times per week × 3 weeks) then practiced at home with video for 50 min sessions 3 times per week × 6 months; control group received educational material related to cognition in a 1 h presentation plus weekly phone call check in	Neurobiological Domain: Synaptic plasticity (BDNF), systemic inflammation (TNF-a & IL-10) secondary outcomes	Cognitive performance with memory (LM), visuospatial ability (Block Design Test), executive function (Digit Span forward-backward, TMT B-A).	TC vs. CG improved memory and executive function (*p* < 0.05), increased plasma BDNF levels (*p* < 0.05). No significance on other outcomes.

Notes: If some study details, such as race, ethnicity, mean age, standard deviation, etc., are not listed, it is because studies did not report that information. Abbreviations: ADAS-Cog = Alzheimer’s Disease Assessment Scale-Cognitive Subscale; ADLs = Activities of Daily Living; AGE = Advanced Glycation End products; a-MCI = amnestic mild cognitive impairment; ANAM-4 = Automated Neuropsychological Assessment Metrics version 4; BBS = Berg Balance Scale; BDNF = brain-derived neurotrophic factor; BNT = Boston Naming Test; BP = Blood pressure; CESD = Center for Epidemiologic Studies Depression Scale; DPA-1 = Dynamic Performance Analysis; DSM-IV = Diagnostic and Statistical Manual of Mental Disorders, Fourth Edition; DSST = Digit Symbol Substitution Test; FES = Falls Efficacy Scale; GNGT = Go/No-go Task of ANAM-4 test; HR = Heart rate; HADS = Hospital Anxiety and Depression Scale; HOMA-IR = Homeostatic Model Assessment of Insulin Resistance; IL-10 = Interleukin-10; LM = Logical Memory Test; MCI = mild cognitive impairment; MDSR = Mattis Dementia Rating Scale; MGT = Matching Grids Task of ANAM-4 test; MMSE = Mini-Mental State Examination; MQ = Wechsler Memory Quotient; MST = Memory Search Task of ANAM-4 test; NPI = Neuropsychiatric Inventory; PRTT = Procedural Reaction Time of ANAM-4 test; PSQI = Pittsburgh Sleep Quality Index; PSS-10 = Perceived Stress Scale; RAVLT = Rey Auditory Verbal Learning Test; ROCF = Rey-Osterrieth Complex Figure Test; sRAGE = soluble receptor of Advanced Glycation End products; T2D = Type 2 Diabetes Mellitus; TC = Tai Chi; TICS-M = Modified Telephone Interview of Cognitive Status; TMT-A = Trail Making Test—Part A; TMT-B = Trail Making Test—Part B; TNF-α = Tumor Necrosis Factor-α; WA = water aerobics; WG = Walking group.

**Table 6:** Included general religious and spiritual activity studies (n = 6).

Study Title	Objective	Authors (Year), Study Location	Study Population Total n (Mean Age +/− SD), % Female, % White; Group (Final n), Demographics	Study Design (CS or L); Time Points	Intervention; Comparison (If Relevant)	Domain: Potential Mechanism (Tool); Mediating Factor or Secondary Outcome	Cognitive Outcome Measure (Tool)	Effects on Outcomes (If Relevant)
Observational: Cross-sectional								
Impact of religious activities on quality of life and cognitive function among elderly	Compare older adult utilization of different types of religious activities in their daily routine and the effects on Quality of Life (QOL) and cognitive function	Amir et al., 2022 Malaysia	n = 432 older suburban adults (60 years+ mean age not reported), 43.5% female, 84.7% Malay, 11.1% Chinese, 3.2% Indian, 0.9% Other	cross-sectional	individual religious activities (obligatory prayers, midnight prayer, recite Al-Quran daily, and fasting).	Psychosocial Domain: QOL (SF-36), depression (GDS-15) Behavioral Domain: instrumental activities of daily living (OARS’s IADL) secondary outcomes	Cognitive function (MoCA)	Overall QOL, depression, ADLs, and cognitive function were significantly higher in participants engaged in religious activities vs. participants less engaged or not practicing at all (*p* < 0.001 to *p* < 0.05 for each comparison).
Older adults with dementia: association of prayer with neuropsychiatric symptoms, cognitive function, and sleep disturbances	Examine the association of private prayer with neuropsychiatric symptoms (NPS), cognitive function, and sleep disturbances in older adults with dementia	Britt et al., 2023 [18] United States (US)	n = 40 older adults with all-cause dementia (84.67 +/− 5.159), 74.9% female, 73.9% non-Hispanic White, 19.2% non-Hispanic Black, 3% Hispanic	cross-sectional with secondary data analysis	private prayer frequency (none, less than once weekly, once per week or more)	Psychosocial Domain: neuropsychiatric symptoms (NPI-Q) Behavioral Domain: sleep disturbances (3 items about sleep) secondary outcomes	Cognitive performance (CDR)	Higher frequency of private prayer was significantly associated with fewer NPS (*p* < 0.01), fewer sleep disturbances (*p* < 0.01), and better cognitive performance (*p* < 0.01).
Association of religious service attendance and neuropsychiatric symptoms, cognitive function, and sleep disturbances in all-cause dementia	Examine the associations between religious service attendance and symptoms of dementia progression	Britt et al., 2023 [16] US	n = 72 older adults with all-cause dementia (84.23 +/− 5.729), 64.5% female, 76.5% non-Hispanic White, 19.8% non-Hispanic Black, 3.7% Hispanic	cross-sectional with secondary data analysis	religious service attendance frequency (none, less than once per week, once a week or more)	Psychosocial Domain: neuropsychiatric symptoms (NPI-Q) Behavioral Domain: sleep disturbances (3 items about sleep) secondary outcomes	Cognitive performance (CDR)	Higher frequency of religious service attendance was significantly associated with fewer NPS (*p* < 0.0005), better cognitive performance (*p* < 0.001) and with fewer sleep disturbances (*p* < 0.0005).
Symptoms of dementia progression in cognitive impairment: the role of religious and spiritual activity	Investigate associations between religious service attendance and NPS, cognition, and sleep disturbances independently at two different time points	Britt et al., 2024 US	n = 63 older adults with mild cognitive impairment (MCI) (81.89 +/− 5.261), 50.1% female, 65.9% non-Hispanic White, 22.1% non-Hispanic Black, 9.9% Hispanic, 2.1% non-Hispanic other	cross-sectional with secondary data analysis 2 time points: baseline and 2 years	religious service attendance frequency (none, less than once per week, once a week or more)	Psychosocial Domain: neuropsychiatric symptoms (NPI-Q) Behavioral Domain: sleep disturbances (3 items about sleep) secondary outcomes	Cognitive performance (CDR)	Higher frequency of religious service attendance was significantly associated with fewer NPS at both time points (*p* < 0.0005 and *p* < 0.0005), better cognitive performance at both time points (*p* < 0.0005 and *p* < 0.0005) and with higher sleep disturbance at baseline (*p* < 0.001) but lower sleep disturbances at time point 2 (*p* < 0.001).
Observational: Longitudinal								
Does religiosity protect against cognitive and behavioral decline in Alzheimer’s dementia?	Identify the relationship between religiosity and the progression of cognitive impairment and behavioral disorders in mild-moderate Alzheimer’s dementia (AD), and the relationship between the patient’s religiosity and caregiver stress	Coin et al., 2010 Italy	n = 64 mild and moderate AD (mean age females 77.8 +/− 6.2; males 73.9 +/− 4.8), 75% Female	longitudinal study 2 time points: baseline, 12 months	religiosity (religious activities frequency of attendance, praying, reading religious materials, and watching/listening to religious programs) [high vs. low], spirituality (personal attitude toward Christianity)	Psychosocial Domain: neuropsychiatric symptoms (NPI) Behavioral Domain: independent and dependent functional abilities (ADL, IADL)	Cognitive performance (MMSE)	Low religiosity was significantly associated with worse cognitive performance (*p* < 0.001), neuropsychiatric symptoms (*p* < 0.001), and decreased functional abilities (*p* < 0.01, *p* < 0.001), while high religiosity showed no significant decline in cognitive performance or neuropsychiatric symptoms; both groups showed significant decline in functional abilities but less so in the higher religiosity group (*p* < 0.05, *p* < 0.01); low religiosity was significantly associated with higher risk of cognitive impairment (OR 6.7).
Quasi-Experimental								
Effect of music intervention on the cognitive and depression status of senior apartment residents in Taiwan	Identify the effect of music Intervention on cognitive function and depression status of residents in senior citizen apartments	Tai et al., 2015 Taiwan	n = 60 older adults n = 41 Experimental, (mean age (80.49 +/− 8.88, 53.7% female); n = 19 Control (mean age 81.68 +/− 4.68, 52.6% female)	quasi-experimental study pretest–posttest comparison 3 time points: Baseline, 1 month, and 4 months	music intervention group using Buddhist hymns	Psychosocial Domain: Depression (GDS-SF) secondary outcome	Cognitive performance (Chinese version MMSE)	4-month cognitive performance significant decline in control group (*p* < 0.028) but no significant change observed in experimental group (*p* < 0.060); 4-month depression scores significantly improved in both groups (experimental group *p* < 0.001; control group *p* < 0.001).

Notes: If some study details, such as race, ethnicity, mean age, standard deviation, etc., are not listed, it is because studies did not report that information. Abbreviations: AD = Alzheimer’s dementia; ADL = Activities of Daily Living; CDR = Clinical Dementia Rating; GDS-15 = 15-item Geriatric Depression Scale; GDS-SF = Geriatric Depression Scale Short Form; IADL = Instrumental Activities of Daily Living; MCI = mild cognitive impairment; MoCA = Montreal Cognitive Assessment; MMSE = Mini-Mental State Examination; NPI-Q = Neuropsychiatric Inventory Questionnaire; NPS = neuropsychiatric symptoms; OARS’s IADL = Older Americans Resources and Services—Instrumental Activities of Daily Living; QOL = quality of life; US = United States.

**Table 7:** Summary comparison of spiritual practices, mechanism pathway, & cognitive outcomes.

Spiritual Practices	Domain	Domain Factors	Cognitive Outcomes
Meditation	Psychosocial	**Depressive symptoms**	**Perceived cognitive impairment and abilities** **Memory** **Processing speed** **Cognitive function & abilities** **Executive function** Verbal fluency Attention & Concentration
		Worry	
		Anxiety	
		**Mood**	
		**Perceived stress**	
		**QOL**	
	Behavioral	**Sleep**	
	Neurobiological	Gene expression	
		**Telomerase activity**	
		**Stress**	
		**Inflammation**	
		**Aβ levels**	
		Cerebral blood flow	
Yoga	Psychosocial	**Perceived stress**	**Visuospatial skills** **Executive function** **Cognitive function** **Memory**
		**Depression**	
		**Psychological distress**	
		**Self-esteem**	
	Neurobiological	Inflammation *	
		Immune response *	
Tai Chi	Psychosocial	**Depression**	**Memory** **Executive function** **Visuospatial processing** **Motor speed and visual attention** **Lower risk of developing dementia** **Cognitive function** **Delayed recall** **Global cognition**
		Anxiety	
		**Perceived stress**	
	Behavioral	**Functional health and balance**	
		**Sleep**	
	Neurobiological	**Glycation**	
		**Synaptic plasticity**	
		**Reaction time in mental shifting**	
General Religious or Spiritual Activities	Psychosocial	QOL	Cognitive function and performance
		Depression	
		Neuropsychiatric symptoms	
	Behavioral	ADLs	
		Sleep	

Notes: **BOLD** indicates evidence from RCT studies; * indicates identified mediator; others are secondary outcomes. Abbreviations: QOL = Quality of life.

**Table 8:** Potential mechanisms to inform the conceptual model.

Psychosocial Domain		
Study	Mediators	Secondary Outcomes
Amir et al., 2022		Depression
		QOL
Britt et al., 2023 [18]		Neuropsychiatric or behavioral symptoms
Britt et al., 2023 [16]		Neuropsychiatric or behavioral symptoms
Britt et al., 2024		Neuropsychiatric or behavioral symptoms
**Eyre et al., 2017**		**Depression**
		**Perceived stress**
**Innes et al., 2018**		**Mood**
		**QOL**
**Lam et al., 2012**		**Depression**
**Lavretsky et al., 2012**		**Depressive symptoms**
		**Mental health**
Lenze et al., 2014		Worry Severity
		Mindfulness
**Milbury et al., 2013**		**Depressive symptoms**
		**Mental health**
Meloni et al., 2013		Anxiety
		Depression
**Oh et al., 2012**		**QOL**
**Sharma et al., 2005**		**Depression**
Spadaro & Hunker et al., 2016		Perceived Stress
**Solianik et al., 2021**		**Depression**
		**Perceived stress**
**Tremont 2022**		**Depression**
		**Perceived stress**
**Behavioral Domain**		
**Study**	**Mediators**	**Secondary Outcomes**
Amir et al., 2022		Activities of Daily Living (ADLs)
Britt et al., 2023 [18]		Sleep
Britt et al., 2023 [16]		Sleep
**Hwang et al., 2020**		**Functional health**
**Innes et al., 2018**		**Sleep**
**Lam et al., 2012**		**Functional balance**
**Nguyen & Kraus, 2012**		**Functional balance**
		**Sleep**
**Solianik et al., 2021**		**Functional health**
**Neurobiological Domain**		
**Study**	**Mediators**	**Secondary Outcomes**
Abomoelak et al., 2023		Gene regulation expression
**Chen et al., 2023**		**Improved fasting glucose levels, glycation end-products**
**Grzenda et al., 2024**	**Inflammatory markers**	
	**Immune response**	
**Innes et al., 2018**		**Stress**
**Lavretsky et al., 2012**		**Telomerase activity**
**Mohan et al., 2011**		**Stress markers and Stress response**
**Oh et al., 2012**		**Inflammatory markers**
Singh et al., 2012		Stress
**Solianik et al., 2012**		**Increased synaptic plasticity**
		**Improved reaction time in mental shifting**
**Sungkarat et al., 2018**		**Increased synaptic plasticity**
Newberg et al., 2010		Increased cerebral blood flow

Note: **BOLD** indicates evidence from RCT studies. Abbreviations: QOL = Quality of life.

## Supplementary Materials

The following supporting information can be downloaded at: https://www.mdpi.com/article/10.3390/brainsci15121296/s1, Table S1: PRISMA completed; Table S2: Database Search; Table S3: Across Practice.

## Abbreviations

The following abbreviations are used in this manuscript:

AD	Alzheimer’s disease
ADRD	Alzheimer’s disease and related dementias
CRP	C-reactive protein
CSF	Cerebral spinal fluid
DMN	Default mode network
fMRI	Functional magnetic resonance imaging
HbA1c	Hemoglobin A1c
IL-6	Interleukin-6
MCI	Mild cognitive impairment
MMAT	Mixed Methods Appraisal Tool
MPIL	Meaning and purpose in life
QOL	Quality of life
RCT	Randomized control trial
