# Supplementary material for: Potential Mechanisms of Influence Between Spiritual Practices and Cognitive Health: A Systematic Review and Conceptual Model
Source: Brain Sci. 2025 Nov 30;15(12):1296. doi: 10.3390/brainsci15121296 (PMC12731188; doi:10.3390/brainsci15121296)
Supplement: Supplementary file 1 [file brainsci-15-01296-s001.zip › Supp Table S2 Additiona Database Search Terms.pdf]

Supplementary Table S2. Additional Five Database Search Terms and Databases

ATLA Terms Table		
**Concepts**	**ATLA Religion Database Subject Headings**	**Keywords**
**Spirituality/Religion**	DE "Spirituality" OR DE "Spirit" DE "Religion" OR DE "Religious attitudes" OR DE "Religiousness"	spirit* OR religio*
**Cognition/Memory**	(DE "Cognition") OR (DE "Memory")	memor* OR cognit* OR "mental health" OR depression OR anxiety OR hopeless OR energy OR appetite OR concentration OR appetite OR concentration OR restless OR nervous OR worry* OR irritable OR afraid
**Support/Meaning**	(DE "Social networks") OR (DE "Human behavior") OR (DE "Stress")	"psychological stress*" OR "physiological stress*" OR anxiety OR depression OR Inflamm* OR immun* OR cortisol OR sleep OR brain OR "brain structure" OR "brain function" OR "brain plasticity" OR neurogenesis OR MRI OR fMRI OR EEG OR ERPs OR ("diffusion sensor" AND imaging) OR BOLD OR VBM OR neurotransmitter OR BDNF OR biomarker OR neuroimmunology OR neurons OR glia OR vasculature OR IGF1 OR VEGF OR hormones OR peptides OR metabolism OR imaging OR neuromodulation OR volumetry OR neuromodulation OR endorphin OR monoamine OR dopamine OR noradrenaline OR norepinephrine OR serotonin OR opioid OR frontal lobes OR parietal lobes OR thalamus OR limbic OR cerebral blood)
**Limits:**	English; 2000-2024	

CINAHL Terms Table		
**Concepts**	**CINAHL Subject Headings**	**Keywords**
**Spirituality/Religion**	(MH "Spirituality") OR (MH "Spiritual Care") OR MH "Religion and Religions+"	spirit* OR religio*
**Cognition/Memory**	(MH "Cognition") OR (MH "Memory") OR (MH “Mental Fatigue”)	memor* OR cognit* OR "problem solving" OR "executive function" OR attention OR "processing speed" OR cognit* OR memory OR "mental health" OR depression OR anxiety OR hopeless OR energy OR appetite OR concentration OR restless OR nervous OR worry* OR irritable OR afraid
**Support/Meaning**	MH "Support, Social+"OR MH "Behavior and Behavior Mechanisms+" OR MH "Stress") OR MH "Life Purpose"	"psychological stress*" OR "physiological stress*" OR anxiety OR depression OR Inflamm* OR immun* OR cortisol OR sleep OR brain OR "brain structure" OR "brain function" OR "brain plasticity" OR neurogenesis OR MRI OR fMRI OR EEG OR ERPs OR ("diffusion sensor" AND imaging) OR BOLD OR VBM OR neurotransmitter OR BDNF OR biomarker OR neuroimmunology OR neurons OR glia OR vasculature OR IGF1 OR VEGF OR hormones OR peptides OR metabolism OR imaging OR neuromodulation OR volumetry OR neuromodulation OR endorphin OR monoamine OR dopamine OR noradrenaline OR norepinephrine OR serotonin OR opioid OR “frontal lobes” OR “parietal lobes” OR thalamus OR limbic OR “cerebral blood” OR support OR stress
**Focus to Core Topic**		TI spirit* OR TI religio*
**Limits**	Jan2020-Dec 2024, English, Adult	

EMBASE Terms Table		
**Concept**	**EMBASE Subject Headings (EMTREE)**	**Keywords**
Spirit/Religion	◎spiritual care◎OR ◎spiritual healing◎ OR ◎religion◎	Spirit* OR religio*
Cognition/Memory	'cognition' OR 'mental performance' OR 'mental fatigue' OR 'memory' OR 'problem solving' OR 'executive function' OR 'attention' OR 'processing speed'	cognit* OR memor* OR "problem solving" OR "executive function" OR attention OR "processing speed" OR cognit* OR memory OR "mental health" OR depression OR anxiety OR hopeless OR energy OR appetite OR concentration OR restless OR nervous OR worry* OR irritable OR afraid
Social Support/Stress	'social support' OR 'mental stress' OR 'physiological stress'	'neuroimmunology' OR neurons OR glia OR vasculature OR igf1 OR vegf OR hormones OR peptides OR metabolism OR imaging OR volumetry OR neuromodulation OR 'endorphin' OR monamine OR dopamine OR 'noradrenalin' OR noradrenoline OR norepinephrine OR 'serotonin' OR opioid OR 'frontal lobes' OR 'parietal lobe' OR thalamus OR limbic OR 'cerebral blood' OR OR 'mental stress' OR 'psychological stress' OR 'physiological stress' OR 'anxiety' OR 'depression' OR inflamm* OR 'inflammation' OR immun* OR cortisol OR 'sleep' OR brain OR 'brain function' OR 'brain structure' OR 'brain plasticity' OR neurogenesis OR mri OR fmri OR eeg OR erps OR ('diffusion sensor' AND imaging) OR bold OR vbm OR 'neurotransmitter' OR bdnf OR biomarker
**Focus on Core Topics**		Spirit*:ti OR Religio*:ti
**Limits:**	Resource: EMBASE, not MEDLINE; Jan 2000-Dec 2024; English; Adults	

PsycInfo Terms Table		
**Concept**	**PsychInfo Subject Headings**	**Keywords**
Spirit/Religion	MAINSUBJECT.EXACT("Religious Practices") OR MAINSUBJECT.EXACT("Religious Beliefs") OR MAINSUBJECT.EXACT("Religion") OR MAINSUBJECT.EXACT("Spiritual Care") OR MAINSUBJECT.EXACT("Spiritual Well Being") OR MAINSUBJECT.EXACT("Spirituality")	Spirit* OR religio*
Cognition/Memory	(MAINSUBJECT.EXACT("Memory") OR MAINSUBJECT.EXACT("Cognitive Assessment") OR MAINSUBJECT.EXACT("Cognitive Aging") OR MAINSUBJECT.EXACT("Cognition")	cognit* OR memor* OR memor* OR cognit* OR "problem solving" OR "executive function" OR attention OR "processing speed" OR cognit* OR memory OR "mental health" OR depression OR anxiety OR hopeless OR energy OR appetite OR concentration OR restless OR nervous OR worry* OR irritable OR afraid
Support/Stress	(MAINSUBJECT.EXACT.EXPLODE("Sense of Purpose") OR MAINSUBJECT.EXACT.EXPLODE("Meaning") OR MAINSUBJECT.EXACT("Behavior") OR MAINSUBJECT.EXACT("Social Support") OR behavior OR support* OR "life purpose"	behavior OR support* OR "life purpose" OR meaning OR "psychological stress*" OR "physiological stress*" OR anxiety OR depression OR Inflamm* OR immun* OR cortisol OR sleep OR brain OR "brain structure" OR "brain function" OR "brain plasticity" OR neurogenesis OR MRI OR fMRI OR EEG OR ERPs OR ("diffusion sensor" AND imaging) OR BOLD OR VBM OR neurotransmitter OR BDNF OR biomarker OR neuroimmunology OR neurons OR glia OR behavior* OR vasculature OR IGF1 OR VEGF OR hormones OR peptides OR metabolism OR imaging OR neuromodulation OR volumetry OR neuromodulation OR endorphin OR monoamine OR dopamine OR noradrenaline OR norepinephrine OR serotonin OR opioid OR "frontal lobes" OR "parietal lobes" OR thalamus OR limbic OR "cerebral blood"
Focus on Core Topics		Title (spirit* OR religio*)
Limits	Date From January 01 2020 to December 31 2024<br>Language: English;<br>Age group: Adulthood (18 yrs & older)	

SocAbs Terms Table		
**Concepts**	**SocAbs Subject Headings**	**Keywords**
**Spirit/Religion**	(MAINSUBJECT.EXACT.EXPLODE("Spirituality") OR MAINSUBJECT.EXACT.EXPLODE("Religion")	Spirit* OR religio*
**Cognition/Memory**	(MAINSUBJECT.EXACT.EXPLODE("Cognition") OR MAINSUBJECT.EXACT("Cognitive functioning") OR MAINSUBJECT.EXACT.EXPLODE("Memory")	cognit* OR memor* OR "problem solving" OR "executive function" OR attention OR "processing speed" OR cognit* OR memory OR "mental health" OR depression OR anxiety OR hopeless OR energy OR appetite OR concentration OR restless OR nervous OR worry* OR irritable OR afraid
**Support/Stress**	(MAINSUBJECT.EXACT.EXPLODE("Meaning") OR MAINSUBJECT.EXACT.EXPLODE("Social support") OR MAINSUBJECT.EXACT.EXPLODE("Support networks") OR MAINSUBJECT.EXACT.EXPLODE("Behavior") OR MAINSUBJECT.EXACT.EXPLODE("Stress")	behavior OR support* OR "life purpose" OR meaning OR "psychological stress*" OR "physiological stress*" OR anxiety OR depression OR Inflamm* OR immun* OR cortisol OR sleep OR brain OR "brain structure" OR "brain function" OR "brain plasticity" OR neurogenesis OR MRI OR fMRI OR EEG OR ERPs OR ("diffusion sensor" AND imaging) OR BOLD OR VBM OR neurotransmitter OR BDNF OR biomarker OR neuroimmunology OR neurons OR glia OR behavior* OR vasculature OR IGF1 OR VEGF OR hormones OR peptides OR metabolism OR imaging OR neuromodulation OR volumetry OR neuromodulation OR endorphin OR monoamine OR dopamine OR noradrenaline OR norepinephrine OR serotonin OR opioid OR "frontal lobes" OR "parietal lobes" OR thalamus OR limbic OR "cerebral blood"
**Focus on Core Topics**		Title (spirit* OR religio*)
**Limits**	Date January 01 2020 to December 31 2024<br>Language: English; Scholarly Journals	
