# Supplementary material for: Potential Mechanisms of Influence Between Spiritual Practices and Cognitive Health: A Systematic Review and Conceptual Model
Source: Brain Sci. 2025 Nov 30;15(12):1296. doi: 10.3390/brainsci15121296 (PMC12731188; doi:10.3390/brainsci15121296)
Supplement: Supplementary file 1 [file brainsci-15-01296-s001.zip › Supp Table S3 across practice.pdf]

Supplementary Table S3. Included studies ordered by practice

Study	Mediating Factors	Secondary Outcomes
**Meditation studies**		
Abomoelak et al., 2023		Gene regulation expression
Innes et al., 2018		QOL, sleep, mood, stress
Lavretsky et al., 2012		Depressive symptoms, telomerase activity, mental health
Lenze et al., 2014		Mindfulness, worry severity
Meloni et al., 2013		Anxiety<br>Depression
Milbury et al., 2013		Depressive symptoms<br>Mental health
Mohan et al., 2011		Stress markers, stress response
Newberg et al., 2010		Increased cerebral blood flow
Oh et al., 2012		QOL, inflammatory markers
Singh et al., 2012		Stress
Spadaro & Hunker et al., 2016		Perceived stress
**Yoga Studies**		
Eyre et al., 2017		Perceived stress, Depression
Grzenda et al., 2024	Inflammatory markers, immune response	
Sharma et al., 2005		Depression
Tremont 2022		Perceived stress, Depression
**Tai Chi Studies**		
Chen et al., 2023		Improved fasting glucose levels, glycation end-products
Hwang et al., 2020		Functional health
Lam et al., 2012		Functional balance, depression
Nguyen & Kraus, 2012		Functional balance, sleep
Port et al., 2018		Anxiety
Solianik et al., 2021		Functional health, depression, increased synaptic plasticity, improved reaction time in mental shifting, perceived stress
Sungkarat et al., 2018		Increased synaptic plasticity
**General R/S Studies**		
Amir et al., 2022		QOL, depression, Activities of Daily Living
Britt et al., 2023a		Neuropsychiatric or behavioral symptoms, Sleep
Britt et al., 2023b		Neuropsychiatric or behavioral symptoms, Sleep
Britt et al., 2024		Neuropsychiatric or behavioral symptoms

Notes. Abbreviations. QOL=Quality of life.
